# Competitive Hydrogen-Bond Partitioning in Deep Eutectic Solvents: From Cooperative Charge Spreading to Structure–Property Design Rules

**DOI:** 10.1021/acsomega.6c02376

**Published:** 2026-06-22

**Authors:** Sergio de-la-Huerta-Sainz, Valentín Diez-Cabanes, Alberto Gutiérrez-Vega, Sara Santamaría, María A. Escobedo, Pedro A. Marcos, Alfredo Bol-Arreba, José L. Trenzado, Mert Atilhan, Santiago Aparicio

**Affiliations:** † Department of Chemistry, 16725 University of Burgos, Burgos 09001, Spain; ‡ International Research Centre in Critical Raw Materials-ICCRAM, University of Burgos, Burgos 09001, Spain; § Department of Physics, University of Burgos, Burgos 09001, Spain; ∥ Department of Physics, University of Las Palmas de Gran Canaria, Las Palmas de Gran Canaria 35017, Spain; ⊥ Department of Chemical and Paper Engineering, 4175 Western Michigan University, Kalamazoo, Michigan 49008-5462, United States

## Abstract

Deep eutectic solvents (DESs) owe their remarkable melting-point depression, high viscosity, and tunable solvation to a hydrogen-bond network far richer than the binary donor–acceptor picture suggests. This study advances the framework of competitive hydrogen-bond partitioning: ionic Cl^–^···H–X interactions, neutral donor–donor self-association, cation-mediated contacts, and water-competitive motifs coexist and continuously redistribute as a function of composition, temperature, and interfacial confinement. Evidence is synthesized from vibrational spectroscopy, multinuclear NMR, neutron and X-ray scattering, dielectric relaxation, classical and ab initio molecular dynamics, DFT cluster calculations, and machine-learning potentials, establishing that no single technique can fully characterize the network—a triangulation criterion requiring at least two independent method categories is essential. A quantitative structure–property framework is developed linking six hydrogen-bond descriptors—motif population, persistence distribution, network connectivity, competitive hydration index, dynamic heterogeneity, and interfacial partitioning—to viscosity, conductivity, diffusion, and glass transition across Type III, Type V, Natural DES (NADES), and hydrophobic DES. A central finding is the cooperativity–mobility tradeoff: cooperative charge spreading at Cl^–^ simultaneously drives eutectic depression and network rigidity, defining a design axis along which DES can be rationally positioned. Water is analyzed as both competitive and cooperative partner across four hydration regimes, and interfacial hydrogen-bond reorganization at electrodes—largely neglected in prior studies—is critically examined. An integrated characterization workflow with standardized reporting criteria, validated force-field benchmarks, and data-driven descriptors for predictive screening is proposed.

## Graphical abstract

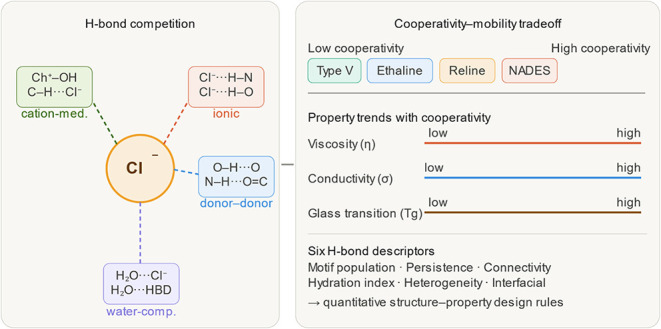

## 1 Introduction

The introduction of choline chloride–urea mixtures as room-temperature liquids by Abbott and coworkers in 2003 established the concept of deep eutectic solvents (DESs) and opened a new chapter in the chemistry of designer solvents.^1^ Pairing choline chloride with carboxylic acids,^2^ polyols,^3^ sugars,^4^ or amino acids^5^ yields low-melting-point mixtures whose properties rival room-temperature ionic liquids at a fraction of the cost and environmental burden. The field has expanded rapidly: more than 10^6^ binary combinations of Hydrogen Bond Acceptot (HBA) and Hydrogen Bond Donor (HBD) are theoretically accessible,^6^ and DESs now find applications in electrodeposition,^7^ biomass processing,^8^ catalysis,^9^ and separations.^10–12^

From the earliest reports, hydrogen bonding has been recognized as the dominant organizing interaction in DESs. The canonical picture attributes eutectic formation to the complexation of chloride by HBD molecules—typically written as [Cl­(HBD)_*x*_]^−^—which delocalizes charge and frustrates crystallization.^30^ This framework proved useful for rationalizing the melting-point depression of simple Type III systems, yet it is now understood to be incomplete. Ashworth, Welton, and Hunt showed by DFT calculations that reline supports an “alphabet soup” of hydrogen-bond types and that doubly ionic motifs (Ch^+^–OH···Cl^–^) can match or exceed the covalency of conventional neutral hydrogen bonds.^13^ Neutron diffraction with isotopic substitution and Empirical Potential Structure Refinement (EPSR) modeling by Hammond, Bowron, and Edler confirmed that all species contribute to a three-dimensional hydrogen-bond network, giving rise to a radially layered structure absent in simple salt solutions.^14^ These findings shifted the conceptual landscape from pairwise Cl^–^···HBD complexation toward a picture in which multiple, competing hydrogen-bond motifs coexist and collectively determine liquid structure.

Landmark syntheses of DES structure–property knowledge^6,7^ and spectroscopic characterization^15,16^ have established the field, and several recent advances make a deeper synthesis timely. In the area of dynamics and heterogeneity, Spittle et al. revealed composition-dependent dynamic domains that decouple from bulk viscosity,^17^ and Chatterjee et al. connected hydrogen-bond rearrangement timescales to local viscosity through ultrafast spectroscopy.^19^ In computation, machine-learning potentials now reproduce DES structure with near-DFT accuracy at classical cost,^18^ and polarizable and first-principles force fields capture charge transfer and cooperativity effects inaccessible to standard fixed-charge models.^22,23^ Regarding network architecture, Stettler et al. showed that a robust hydrogen-bonded network emerges well below the canonical eutectic composition,^20^ Pietropaoli et al. demonstrated that Type V DES require a fundamentally different bonding paradigm involving π–π interactions,^21^ and Pandian et al. systematically linked diol chain length to network architecture and transport.^24^ Despite this extensive body of work, no study has yet attempted a unified, quantitative treatment that (i) treats hydrogen bonding as a competitive, multimotif phenomenon, (ii) links hydrogen-bond descriptors systematically to macroscopic properties, and (iii) addresses the underreported interfacial and cooperative dimensions of the network.

**Table 1:** Experimental and Computational Methods for Hydrogen-Bond Characterization in Deep Eutectic Solvents: Observables, Capabilities, and DES-Specific Considerations

Method	Category	Primary HB observables	Accessible time scale/length scale	Static vs dynamic	Key strengths	Limitations and DES-specific pitfalls	Recommended pairing
FTIR/ATR-IR	Spectroscopic	O–H, N–H stretching shifts; band broadening; low-frequency intermolecular modes	Instantaneous (vibrational); bulk average	Static (time-averaged)	Fast acquisition; donor-specific compositional trends; in situ temperature scans	Band overlap between multiple HBD modes; ambiguous assignment without independent support; hygroscopicity of DES complicates ATR sample preparation; quantitative deconvolution often underdetermined	NMR, MD, Raman
Raman/UVRR	Spectroscopic	Intermolecular modes; donor local environments; lattice-like bands; C–H and O–H environments	Instantaneous; local (∼0.5 nm)	Static	Complementary selection rules to IR; sensitive to local symmetry; in situ and operando capability; suitable for aqueous DES	Mode assignment can be nonunique; fluorescence interference in some HBD systems; resonance Raman requires UV source access	AIMD (for mode assignment), DFT clusters
^1^H/^35^Cl NMR	Spectroscopic	Chemical shift perturbations; exchange averaging; Cl^–^ coordination environment; H-bond strength proxies	μs–ms exchange; local electronic	Dynamic (exchange-averaged)	Direct local electronic probes; ^35^Cl uniquely sensitive to Cl^–^ H-bond environment; composition-resolved; detects competitive hydration	Broad linewidths in viscous neat DES (η > 200 mPa·s); ^35^Cl quadrupolar relaxation limits resolution; temperature calibration critical in viscous media	DOSY, MD
DOSY NMR	Spectroscopic	Self-diffusion coefficients; species-resolved mobility; dynamic heterogeneity proxies	ms–s (diffusion time); ∼nm displacement	Dynamic	Transport-sensitive; composition-resolved; distinguishes mobility of each species independently	Interpretation indirect for H-bond counts; convection artifacts in low-viscosity samples; gradient calibration required; does not directly probe H-bond geometry	Viscosity, MD, conductivity
Neutron/X-ray scattering + EPSR	Structural	Pair correlation functions *g*(*r*); coordination numbers; nanoscale ordering (S(Q))	Instantaneous structure; 0.1–10 nm	Static (ensemble average)	Most direct structural constraints; H/D contrast in neutron; EPSR provides atomistic 3D models	Facility access required; isotopic substitution costly; EPSR models not unique; requires large sample volumes; modeling complexity for multicomponent DES	MD/AIMD for validation
Dielectric spectroscopy	Spectroscopic	Dipolar relaxation times; dc conductivity; relaxation strength; Cole–Cole parameters	ps−μs (frequency domain)	Dynamic	Broad dynamic range; connects relaxation to transport; sensitive to glassy dynamics and ion pairing	Deconvolution of overlapping relaxation modes complex; electrode polarization at low frequencies; interpretation model-dependent	MD, viscosity, NMR
DSC/TGA	Thermal	Glass transition (Tg); melting/eutectic temperature; thermal decomposition; phase behavior	Equilibrium/kinetic	Equilibrium (static)	Routine screening; detects phase separation and eutectic formation; quantifies thermal stability	Does not directly probe H-bond geometry or dynamics; heating rate affects Tg values; moisture uptake during measurement if not sealed; limited mechanistic information	DFT (energetics), MD
Sum frequency generation (SFG)	Advanced/Surface	Interfacial vibrational spectra; molecular orientation at surfaces; surface speciation	Instantaneous; topmost ∼1 nm	Static (surface)	Surface-selective; identifies interfacial molecular orientation and speciation changes with hydration or potential	Requires optical access and flat interfaces; signal interpretation needs modeling; limited to accessible interfaces; not routine	Interfacial MD, XPS
Classical MD	Computational	RDFs; H-bond counts, angles, lifetimes; cluster distributions; MSD; coordination numbers	ps−μs; 1–20 nm	Both	Atomistic dynamics across ns timescales; direct access to H-bond geometry, lifetime distributions, and cluster statistics; composition and temperature scans feasible	Force-field dependence is critical: nonpolarizable FF may miss charge transfer and cooperativity; H-bond definition (distance/angle cutoff) affects counts by 20–40%; charge scaling choice (0.8 vs 1.0) strongly impacts dynamics	Experiment (spectroscopy, transport) for validation
AIMD/DFT-MD	Computational	Electronic structure; charge redistribution; short-time H-bond dynamics; proton transfer propensity; vibrational spectra from DACF	fs–ps; 1–2 nm	Both	High fidelity for bonding, polarization, and charge transfer; no empirical H-bond definition needed; can compute IR/Raman spectra directly	Small simulation cells (100–300 atoms); short trajectories (10–100 ps); computationally expensive; statistical sampling limited; finite-size effects on dynamics	Raman/IR (spectra), NMR (shifts), classical MD (validation)
DFT cluster calculations	Computational	Relative motif energies; interaction energy decomposition; vibrational frequency assignments; NBO/QTAIM analysis	N/A (static minima)	Static	Mechanistic interpretation of local H-bond motifs; quantitative energy ranking; NBO analysis reveals charge transfer	No liquid-state statistics or dynamics; gas-phase or implicit-solvent models miss many-body and entropic effects; basis set superposition error; cluster size selection bias	Raman/IR, AIMD
Machine learning potentials	Computational (advanced)	Near-AIMD accuracy for structure and dynamics at classical MD cost; H-bond populations and lifetimes	ps–ns; 2–10 nm	Both	Combines electronic-structure accuracy with long trajectories and large cells; transferable across compositions	Requires extensive AIMD training data; validation against experiment still sparse for DES; overfitting risk; not yet standardized for DES force fields; limited availability of pretrained models	AIMD (training), experiment (validation)

**Figure 1:**
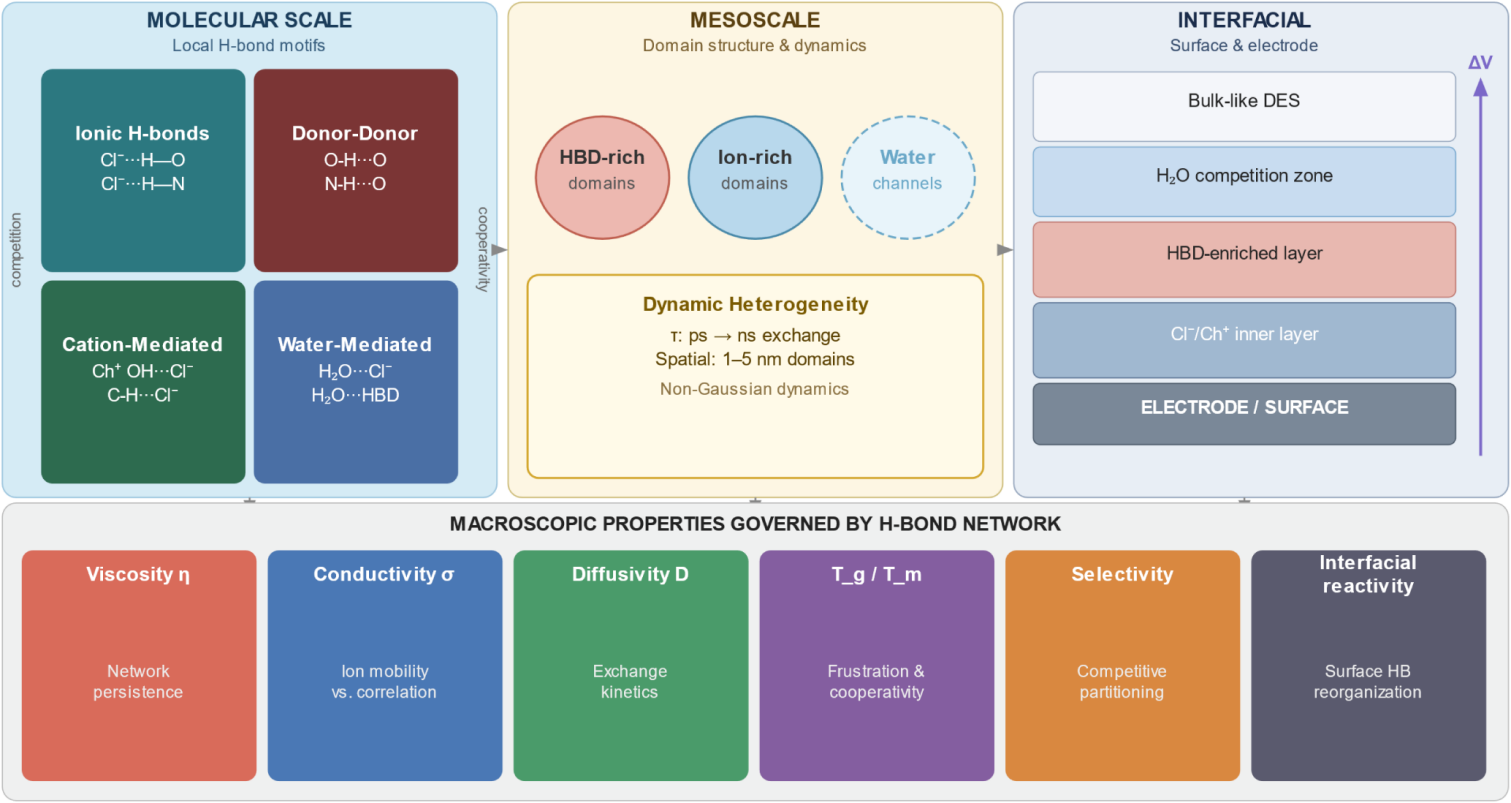
Multiscale hydrogen-bond organization in deep eutectic solvents. The left panel illustrates the four primary classes of local hydrogen-bond motifs: ionic (Cl^–^···H–O, Cl^–^···H–N), donor–donor (O–H···O, N–H···O), cation-mediated (Ch^+^ OH···Cl^–^, C–H···Cl^–^), and water-mediated (H_2_O···Cl^–^, H_2_O···HBD). These motifs are linked by competition and cooperation, generating mesoscale features (center) including HBD-rich and ion-rich nanodomains, water channels, and dynamic heterogeneity with characteristic lifetimes spanning picoseconds to nanoseconds and spatial scales of 1–5 nm. The right panel depicts the interfacial regime at an electrode or solid surface, showing the layered restructuring of Cl^–^/Ch^+^, HBD, and water relative to the bulk. The bottom panel connects the hydrogen-bond network architecture to the macroscopic properties it governs: viscosity (η), ionic conductivity (σ), self-diffusion (*D*), glass transition and melting temperatures (Tg/Tm), selectivity, and interfacial reactivity.

**Figure 2:**
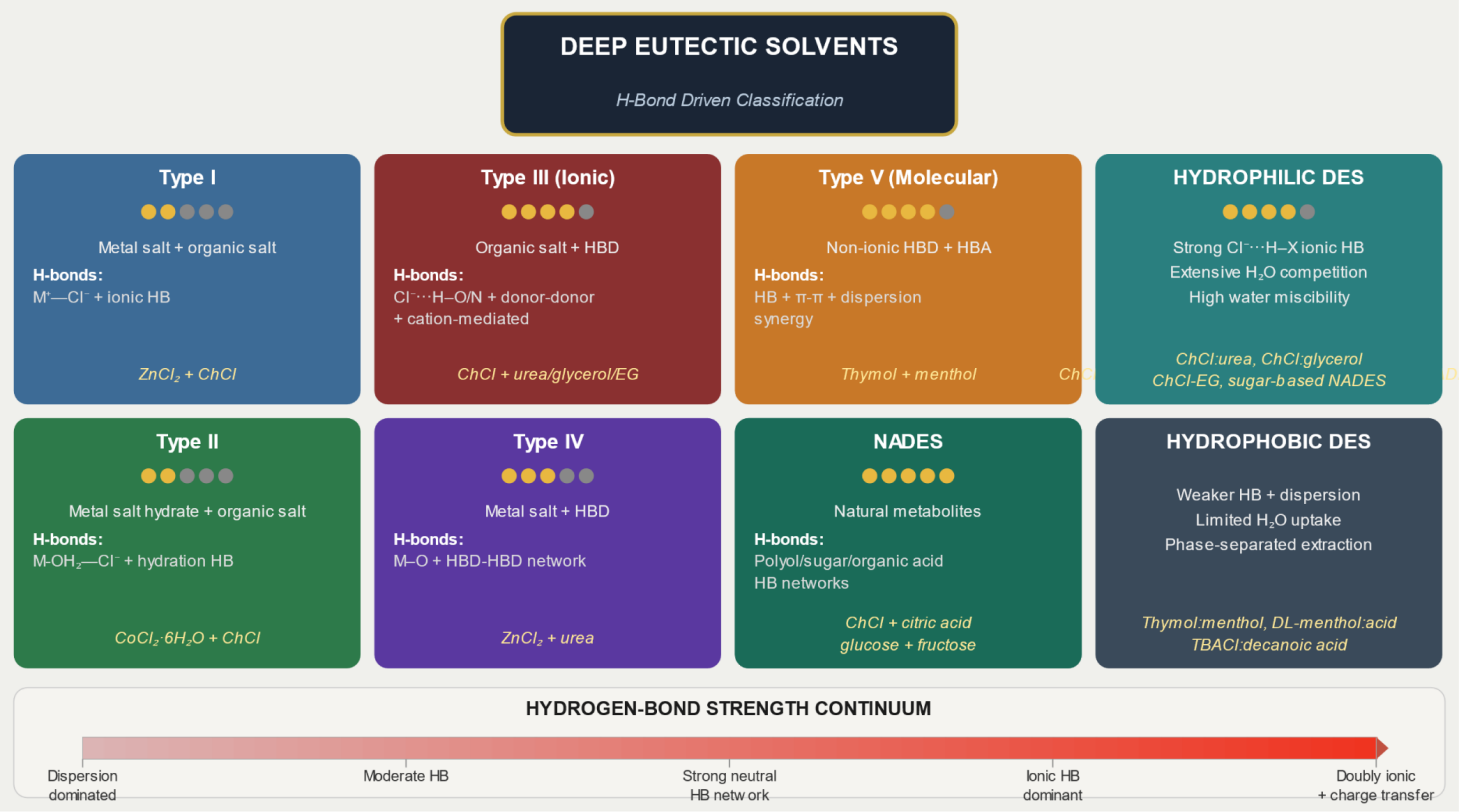
Hydrogen-bond-driven classification of deep eutectic solvents. Each DES type is presented with its general composition, dominant hydrogen-bond motifs, representative examples, and a qualitative indicator (filled circles, ●) of the relative hydrogen-bond contribution to eutectic formation. Type III (ionic, organic salt + HBD) exhibits the strongest H-bond involvement through Cl^–^···H–O/N ionic interactions and donor–donor self-association. Type V (molecular, nonionic HBD + HBA) relies on a synergy of neutral O–H···O hydrogen bonds, π–π stacking, and dispersion. NADES (natural DES) feature multifunctional donors (polyol/sugar/organic acid) with complex, multisite hydrogen-bond networks. Hydrophilic and hydrophobic DES are distinguished by their water interactions: extensive Cl^–^-centered competitive hydration versus limited H_2_O uptake with dispersion-dominated cohesion. The bottom bar illustrates the hydrogen-bond strength continuum across DES types, ranging from dispersion-dominated (Type V) to doubly ionic with charge transfer (Type III).

**Figure 3:**
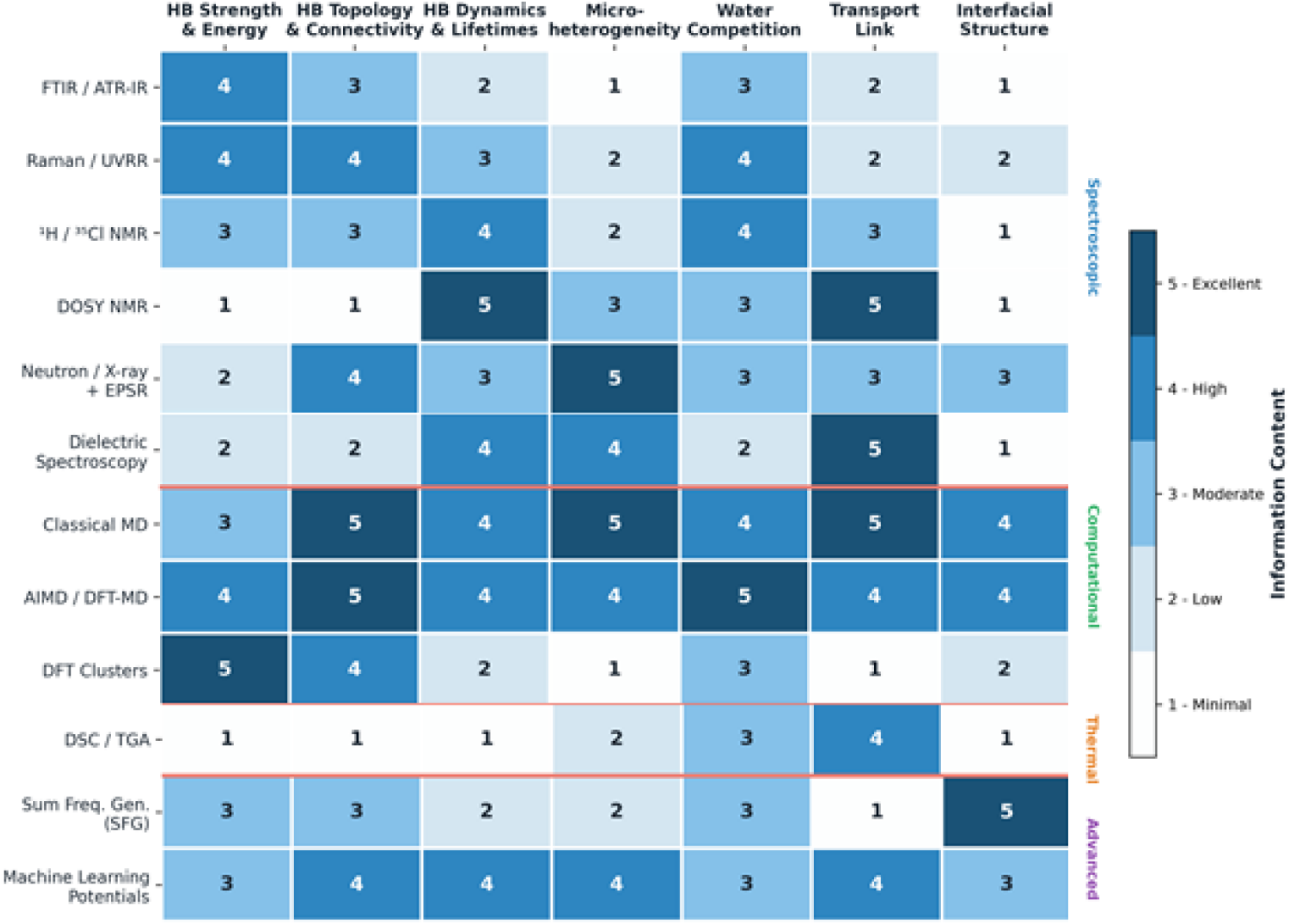
Semiquantitative, expert-assessed information-content matrix for experimental and computational methods used in DES hydrogen-bond characterization. Each cell indicates the expert-assessed information content (1 = no useful information; 2 = low/indirect; 3 = moderate, ensemble-averaged; 4 = high quality with specific limitations; 5 = state-of-the-art, comprehensive) of a given technique for probing a specific hydrogen-bond property; see Table 7, . Methods are grouped into spectroscopic (FTIR/ATR-IR, Raman/UVRR, ^1^H/^35^Cl NMR, DOSY NMR), structural (neutron/X-ray scattering with EPSR), advanced experimental (dielectric spectroscopy, sum frequency generation), thermal (DSC/TGA), and computational (classical MD, AIMD/DFT-MD, DFT clusters, machine learning potentials) categories. The scores reflect comparative literature assessment and expert consensus; they are not derived from directly measured thresholds and should be treated as structured guidance rather than objective rankings. The matrix highlights that no single technique provides complete hydrogen-bond characterization: classical MD and AIMD score highest across multiple properties, while spectroscopic methods excel at strength and water competition but have limited access to microheterogeneity and interfacial structure. Optimal characterization requires combinations spanning at least two categories to satisfy the triangulation criterion discussed in Section 2.

**Figure 4:**
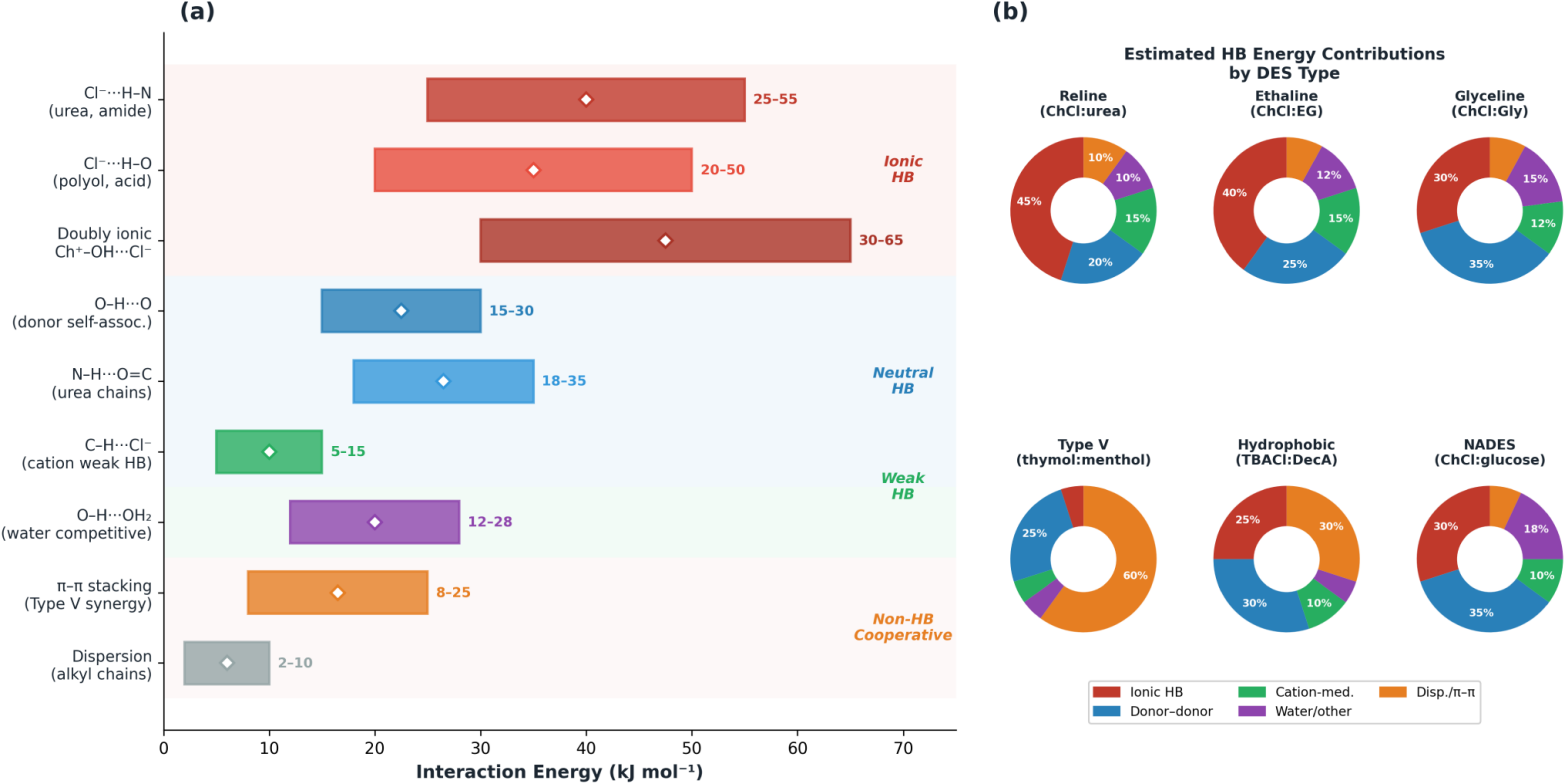
Hydrogen-bond energy landscape in deep eutectic solvents. (a) Interaction energy ranges (kJ mol^–1^) for the principal hydrogen-bond and noncovalent motifs encountered in DES, organized by class: ionic H-bonds (Cl^–^···H–N, Cl^–^···H–O, doubly ionic Ch^+^–OH···Cl^–^), neutral H-bonds (O–H···O donor self-association, N–H···O=C urea chains), weak H-bonds (C–H···Cl^–^ cation contributions), water-competitive motifs (O–H···OH_2_), and non-H-bond cooperative interactions (π–π stacking, dispersion). Diamond markers indicate estimated midpoints, and bars represent the ranges reported in computational and spectroscopic literature. (b) Estimated hydrogen-bond energy contributions by motif type for six representative DES systems: reline, ethaline, glyceline, Type V (thymol:menthol), hydrophobic (TBACl:decanoic acid), and NADES (ChCl:glucose). Donut charts show the approximate fractional energy contribution of ionic H-bonds (red), donor–donor (blue), cation-mediated (green), dispersion/π–π (orange), and water/other (purple) interactions. Values are synthesized from representative literature and should be interpreted as comparative trends rather than absolute measurements.

**Table 2:** Representative DES Systems: Composition, Hydrogen-Bond Characteristics, and Physicochemical Properties^a^

DES system	Type	HBA:HBD (molar ratio)	Dominant H-bond motifs	Key characterization evidence	η at 298 K (mPa·s)	σ at 298 K (mS cm^–1^)	Tg (K)	Avg. Cl^–^coord. number	Water sensitivity
Reline (ChCl:urea)	III	1:2	Cl^–^···H–N, Cl^–^···H–O (ionic); urea–urea N–H···O=C self-association; Ch^+^ C–H···Cl^–^	AIMD, Raman/IR, neutron diffraction + EPSR, NMR, classical MD	∼750	∼0.2	∼205	3.5–4.5	Strong: competitive hydration at 5–30 wt% H_2_O sharply reduces η
Ethaline (ChCl:EG)	III	1:2	Cl^–^···H–O (ionic); EG O–H···O network; Ch^+^- mediated	Neutron + MD + AIMD, transport, dielectric spectroscopy	∼36	∼7.6	∼170	3.0–4.0	Moderate: gradual η decrease; conductivity nonmonotonic
Glyceline (ChCl:glycerol)	III	1:2	Glycerol self-association dominates collective dynamics; Cl^–^···H–O present but secondary	JPCB spectroscopy/dynamics, QENS, dielectric, classical MD	∼376	∼1.1	∼190	3.0–3.8	Strong: OH-rich donor enables extensive water competition
ChCl:malonic acid	III	1:1	Cl^–^···H–O(COOH); acid dimer motifs; strong donor self-association	IR, NMR, MD	∼721	∼0.6	∼195	3.0–3.5	High: acid–water competition disrupts dimer network
ChCl:oxalic acid	III	1:1	Cl^–^···H–O(COOH); intramolecular COOH···COOH; limited donor flexibility	IR, Raman, MD	∼3500	∼0.1	∼200	2.5–3.5	High
ChCl:polyols (series)	III	1:2 to 1:4	Cl^–^···H–O donor-dependent; donor architecture (chain length, OH spacing) tunes network	FTIR + ^1^H/^35^Cl NMR systematic series	Variable (36–750+)	Variable	Variable	3.0–4.5	Composition-dependent
ChCl:citric acid (NADES)	III/NADES	1:1	Multifunctional donor: 3 × COOH + 1 × OH; extensive intra/intermolecular HB; Cl^–^ coordination complex	Raman, NMR, MD	∼5000+	∼0.05	∼210	2.5–3.5	Very high: water integral to NADES structure
ChCl:glucose (NADES)	III/NADES	1:1 (+ water)	Multiple OH groups; extensive donor self-association; water often structural component	DSC, IR, MD	∼8000+ (neat)	<0.05	∼215	3.0–4.0	Water is structural, not just additive
Thymol:menthol	V	1:1	Neutral O–H···O only; π–π/C–H···π synergy; packing entropy contribution; no ionic HB	Thermal analysis, Raman, MD	∼25	∼0.01 (nonionic)	∼155	N/A	System-specific; limited water uptake
Hydrophobic: dl-menthol:decanoic acid	V/Hydrophobic	Various	Acid O–H···O dimers; O–H···O(menthol); dispersion dominant; sparse HB network	NMR, COSMO-RS, MD	∼15–50	<0.01	∼145	N/A	Very high even at trace moisture: disrupts acid dimers
TBACl:decanoic acid (hydrophobic, ionic)	Hydrophobic (ionic)	1:2	Acid dimers + Cl^–^···H–O(acid) ionic motifs; amphiphilic aggregation	Multinuclear NMR + MD	∼180	∼0.3	∼180	2.0–3.0	Very high: 1–3 wt% H_2_O alters ion pairing and phase behavior
Reline + 20 wt% H_2_O	III (hydrated)	1:2 + water	H_2_O···Cl^–^ competitive; reduced urea–Cl^–^ persistence; new H_2_O-mediated exchange pathways	AIMD, NMR, IR, MD	∼30–50	∼5–10	∼155	2.5–3.5 (Cl^–^ partially hydrated)	N/A (hydrated state)

a Viscosity, conductivity, and Tg values are representative ranges from the cited literature at or near 298 K and atmospheric pressure. Exact values depend on water content, preparation protocol, and measurement technique. Cl^–^ coordination numbers are estimated from MD/AIMD simulations and reflect the average number of HBD hydrogen atoms within the first coordination shell.

**Figure 5:**
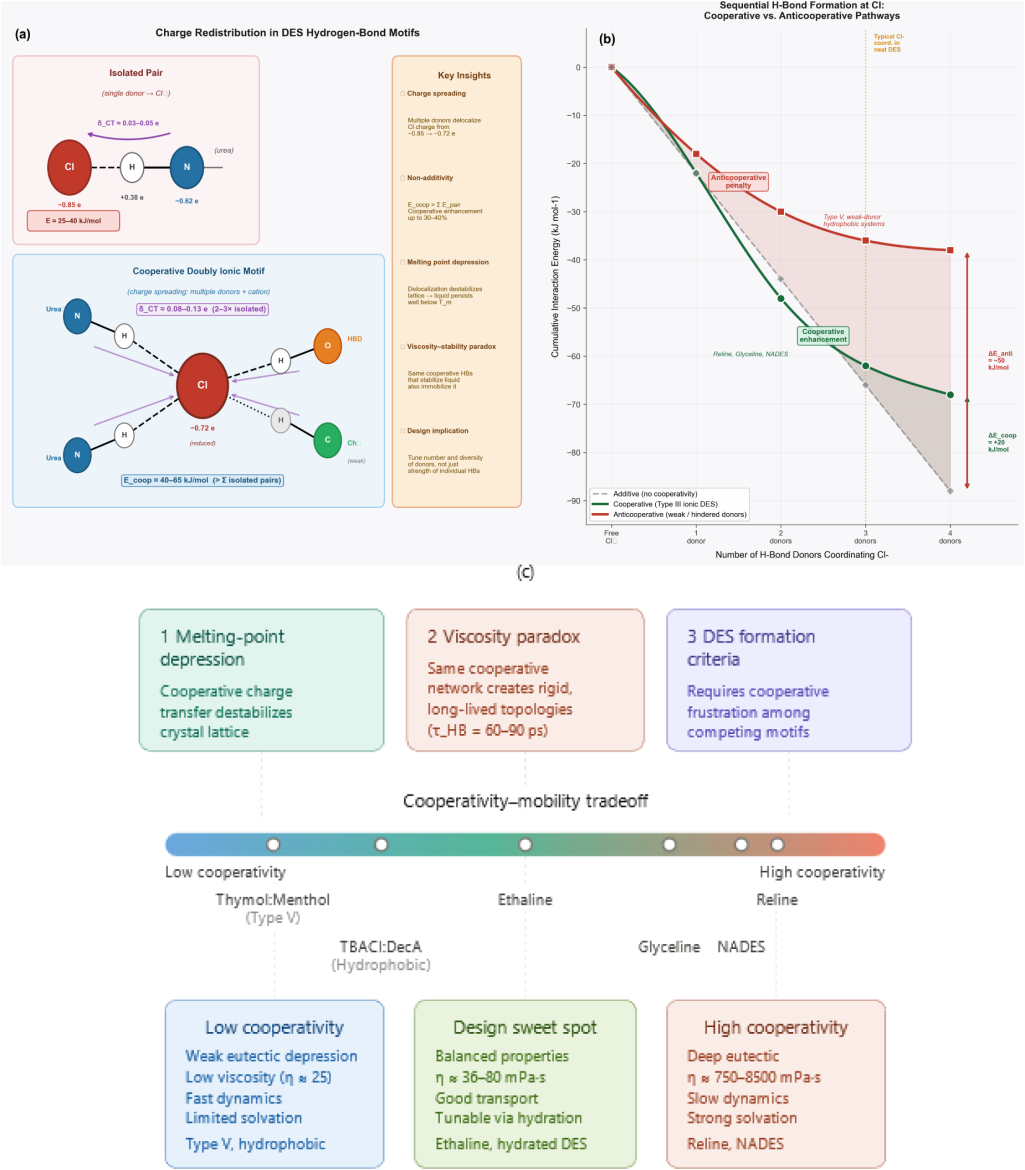
Charge spreading, cooperative hydrogen bonding, and their consequences for DES formation and properties. (a) Comparison of charge redistribution in an isolated pairwise Cl^–^···H–N­(urea) interaction (left) versus a cooperative, doubly ionic environment in which Cl^–^ is coordinated by multiple hydrogen-bond donors (right). In the isolated motif, charge transfer from the donor to Cl^–^ is modest (Δ*q* ≈ 0.03 e, NBO analysis,^41^ E_HB ≈ −35 kJ mol^–1^). In the full solvation shell, cumulative charge transfer reaches ΣΔ*q* ≈ 0.13 e, NBO analysis,^13,41^ and the total interaction energy (ΣE_HB ≈ −160 kJ mol^–1^) exceeds the sum of isolated pairwise contributions, demonstrating positive cooperativity. The reduced effective charge on Cl^–^ (from −0.85 e to −0.72 e, NBO analysis^41^) reflects the charge-spreading mechanism proposed by Zahn and Kirchner, which simultaneously stabilizes the liquid state and stiffens the hydrogen-bond network. (b) Energy diagram illustrating cooperative versus anticooperative hydrogen-bond addition to Cl^–^ as successive HBD molecules enter the first coordination shell. In the cooperative regime (teal, reline-like systems), each additional donor contributes a progressively larger stabilization increment (−35, −42, −45 kJ mol^–1^, estimated from DFT cluster calculations in refs 13,31,41 ), exceeding the additive reference (gray dashed). In the anticooperative regime (magenta), steric crowding or electrostatic saturation causes diminishing returns (−28, −18, −12 kJ mol^–1^, representative estimates, refs 13,31,41 ). These values represent net many-body interaction energy increments and include donor–donor contributions alongside the Cl^–^···H–X bond; they are qualitative descriptors of the cooperative vs anticooperative trend rather than bond-decomposed quantities (see Section 3.3 for discussion). The cooperative path leads to deep eutectic depression coupled with high viscosity; the anticooperative path explains why some HBA/HBD combinations with strong pairwise hydrogen bonds fail to form stable DES. (c) Three principal consequences of charge spreading and cooperativity for DES behavior: (1) melting point depression—cooperative charge transfer destabilizes the crystal lattice, producing the characteristic large ΔTm; (2) the viscosity paradox—the same cooperative network that depresses Tm creates persistent, rigid hydrogen-bond topologies with long lifetimes (τ_HB), explaining why DES such as reline combine very low melting points with viscosities of ∼750 mPa·s; (3) DES formation criteria—successful eutectic formation requires not just strong pairwise interactions but sufficient cooperative frustration among multiple competing motifs to suppress crystallization. The bottom panel presents the cooperativity–mobility tradeoff as a design axis: low-cooperativity systems (Type V, hydrophobic DES) offer fast dynamics but weak eutectic depression and limited solvation, while high-cooperativity systems (reline, NADES) achieve deep eutectics and strong solvation at the cost of transport. The design sweet spot for most applications lies at moderate cooperativity (e.g., ethaline, hydrated DES), where eutectic stability and molecular mobility are balanced. Partial charge values and energy increments are representative estimates consistent with DFT and AIMD literature. Note that alternative charge-partitioning schemes (QTAIM, Hirshfeld, CM5) yield numerically smaller charge-transfer values while supporting the same qualitative cooperative trend, so absolute figures should be treated as order-of-magnitude guides.

**Figure 6:**
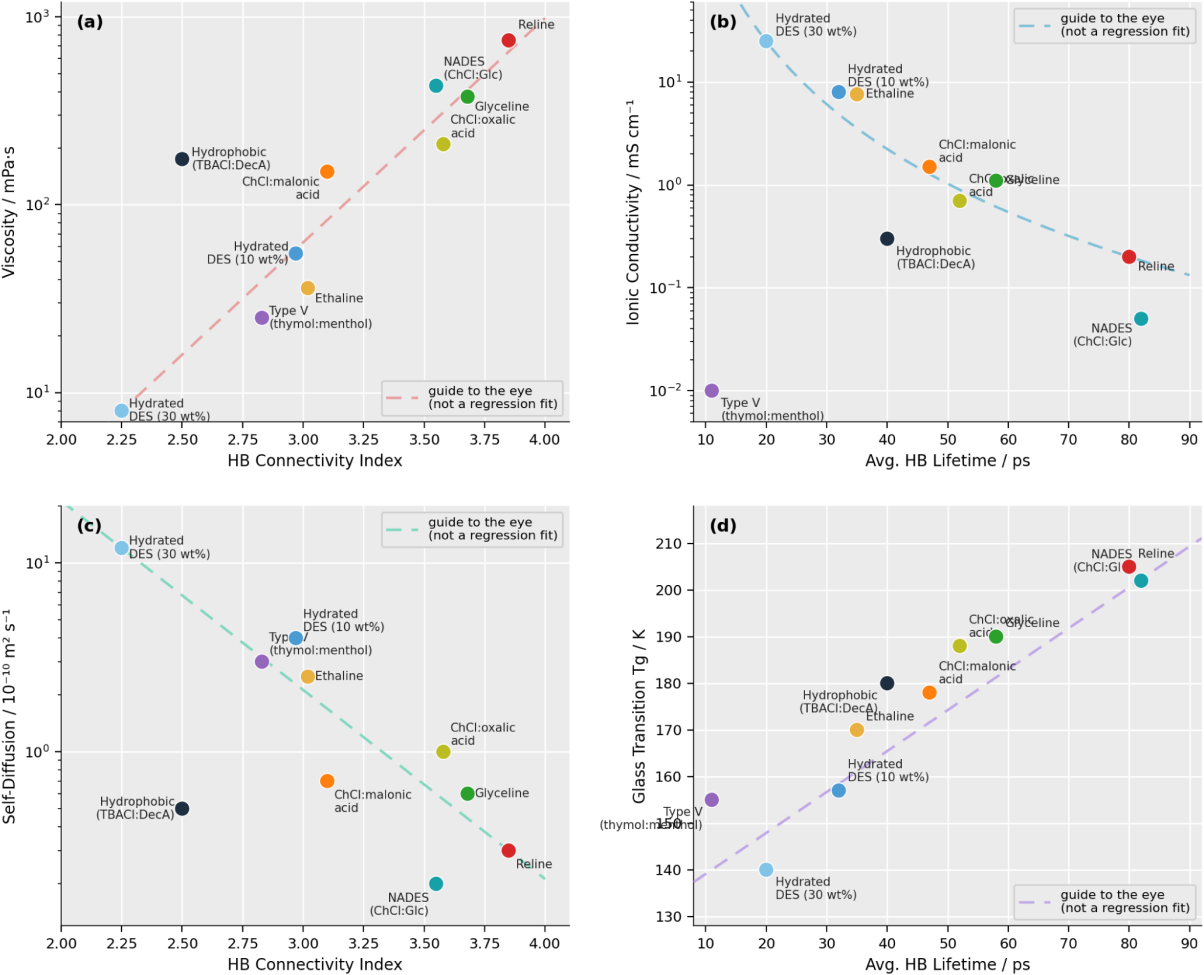
Structure–property correlations linking hydrogen-bond network descriptors to macroscopic DES properties. (a) Viscosity (η, mPa·s, logarithmic scale) versus normalized hydrogen-bond connectivity index, showing an approximately exponential increase with network connectivity across DES types. (b) Ionic conductivity (σ, mS cm^–1^, logarithmic scale) versus average hydrogen-bond lifetime, illustrating the inverse relationship between H-bond persistence and ion mobility. (c) Self-diffusion coefficient (*D*, 10^–10^ m^2^ s^–1^) versus hydrogen-bond connectivity index, demonstrating exponential decay as the network becomes more connected. (d) Glass transition temperature (Tg, K) versus average hydrogen-bond lifetime, showing a positive linear correlation between H-bond persistence and vitrification tendency. Each data point represents a different DES system at 298 K (or its representative operating temperature). Dashed lines are trend guides. Hydrated DES (10 and 30 wt% water) are included to illustrate how water shifts systems along the correlation by reducing connectivity and persistence. Data are synthesized from representative literature values and normalized conceptual descriptors.

**Table 3:** Proposed Descriptor Set for Hydrogen-Bond-Informed DES Screening and Design^a^

Priority	Descriptor	Operational definition	How to measure/estimate	Representative values (reline **→** ethaline)	Current maturity	Why it matters
1	HB motif population	Fractional distribution of Cl^–^···H–O, Cl^–^···H–N, donor–donor, cation-mediated, and water-mediated motifs	MD/AIMD radial and angular distribution analysis + spectral deconvolution (IR, Raman)	Reline: ∼45% ionic, ∼20% donor–donor, ∼15% cation, ∼10% water; Ethaline: ∼40% ionic, ∼25% donor–donor, ∼12% cation, ∼15% water	Routine (MD); moderate (experiment)	Captures the chemistry of local interactions; distinguishes DES types; determines which motifs dominate structure
2	HB persistence distribution	Survival probability *C*(*t*) or lifetime spectrum of each motif class; characterized by mean lifetime τ_HB and stretching exponent β	MD time-correlation functions; NMR exchange rate proxies (T_1_, T_2_, DOSY); dielectric relaxation	Reline: τ_HB ≈ 60–90 ps (Cl^–^···H–N), β ≈ 0.55; Ethaline: τ_HB ≈ 25–40 ps, β ≈ 0.65	Routine (MD); emerging (experiment)	Separates strong–static from labile networks; directly correlates with viscosity and diffusion; explains transport more than average counts
3	Network connectivity index	Graph-theory metric: average degree, clustering coefficient, and percolation threshold of the instantaneous H-bond network	Graph analysis on MD trajectories (nodes = molecules, edges = H-bonds); percolation analysis	Reline: high connectivity (avg. degree ∼3.8, percolating); Ethaline: moderate (∼3.0); Hydrated DES: reduced (∼2.3)	Emerging	Links directly to viscosity and heterogeneity; captures network topology beyond pairwise counts; predicts gel-like vs fluid behavior
4	Competitive hydration index	Fractional shift in motif populations (Δ_ionic, Δ_donor–donor) upon addition of water from 0 to 10 wt%	Hydration series: spectroscopy (IR/NMR shifts) + MD motif counting at fixed *T*	Reline: Δ_ionic ≈ −15% at 10 wt% H_2_O; Hydrophobic DES: Δ_ionic ≈ −60% at 3 wt%	Emerging	Predicts water sensitivity and process robustness; essential for applications where moisture control is impractical
5	Dynamic heterogeneity metric	Non-Gaussian parameter α_2_(*t*) at its maximum; alternatively, ratio of fast to slow diffusion components; or distribution width of local relaxation times	Time-resolved spectroscopy (solvation dynamics); MD: α_2_(*t*), van Hove function; dielectric spectroscopy: Cole–Cole α	Reline: α_2_,max ≈ 1.5–2.5; Ethaline: α_2_,max ≈ 0.8–1.2; Hydrated DES: α_2_,max ≈ 0.3–0.6	Emerging (MD routine; experiment early)	Critical for understanding transport anomalies; explains why averaged descriptors often fail to predict properties; growing JPC B focus area
6	Interfacial HB partitioning	Difference in motif populations and orientation order parameters between the bulk (>3 nm from surface) and interfacial (<1 nm) regions	Surface-sensitive spectroscopy (SFG, XPS) + interfacial MD; potential-dependent studies	Reline at Pt: Cl^–^ enrichment ∼2× bulk density at IHL; HBD depletion ∼40%	Aspirational (few studies available)	Required for electrochemistry and catalysis; interfacial HB topology controls electrode kinetics and selectivity; grossly underreported

a Representative values are synthesized from the cited literature and should be used for comparative guidance rather than as absolute benchmarks. Priority ranking reflects the recommended order of implementation for the DES characterization studies.

**Figure 7:**
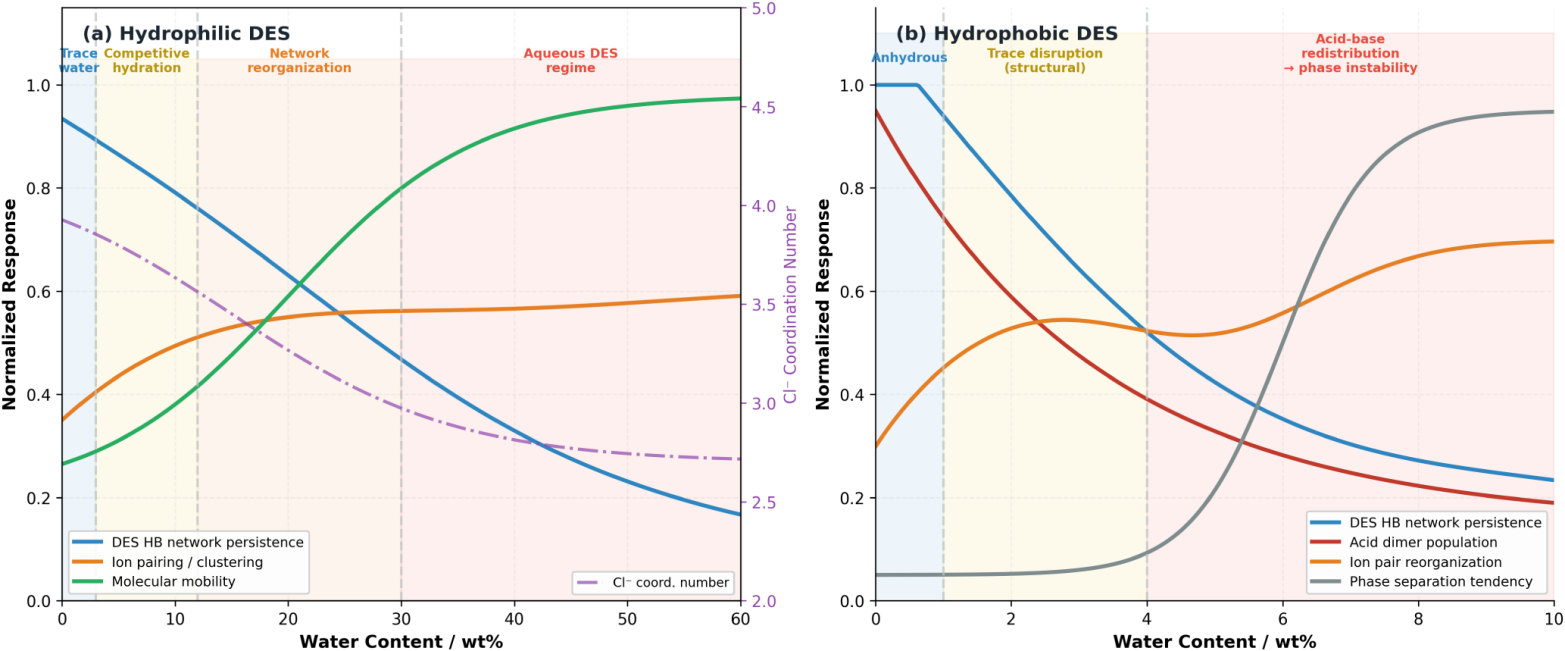
Hydration regime maps for hydrophilic and hydrophobic deep eutectic solvents. (a) Hydrophilic DES (e.g., ChCl-based systems): normalized response curves for DES hydrogen-bond network persistence (blue), ion pairing/clustering (orange), molecular mobility (green), and Cl^–^ coordination number (purple, right axis) as a function of water content (0–60 wt%). Four regimes are indicated: trace water (<3 wt%, [≈<31 mol%]), competitive hydration (3–15 wt%, [≈31–72 mol%]), network reorganization (15–30 wt%, [≈72–86 mol%]), and the aqueous DES regime (>30 wt%). (b) Hydrophobic DES (e.g., TBACl:decanoic acid): normalized response curves for H-bond network persistence (blue), acid dimer population (red), ion pair reorganization (orange), and phase separation tendency (gray) over a much narrower water range (0–10 wt%, [≈0–79 mol%, relative to the TBACl:DecA formula unit ≈ 622 g mol^–1^]), reflecting the heightened moisture sensitivity of hydrophobic systems. Note the sharp structural disruption at 1–3 wt% (≈26–52 mol%) water. Curves represent normalized conceptual trends synthesized from representative literature; regime boundaries are approximate and system-dependent.

**Table 4:** Recommended Minimum Reporting Standards for Hydrogen-Bond-Focused DES Studies

Priority issue	Criticality	Current common practice	Specific recommended reporting	Rationale
Water content	Essential (all studies)	Often stated qualitatively (“dried” or “anhydrous”) or omitted entirely	Report wt% and/or mol% by Karl Fischer titration or TGA; specify timing (before, during, after measurement); report method sensitivity (±ppm)	Even 0.5 wt% H_2_O can shift viscosity by 10–20% and alter Cl^–^ coordination; unreported moisture is the single largest source of irreproducibility
Water content units	Strongly recommended	wt% only; mol% rarely reported	Report both wt% and mol%, specifying the formula-unit molar mass used as the basis for the mol% calculation (e.g., ChCl·2urea ≈ 260 g mol^–1^ for reline)	The wt% and mol% scales diverge dramatically for DES/water mixtures (e.g., 10 wt% H_2_O in reline ≈ 62 mol%), obscuring the true compositional regime; dual reporting enables thermodynamically consistent cross-study comparison
Sample preparation and equilibration	Essential	Mixing time, temperature, and atmosphere rarely specified	Report mixing temperature and duration; equilibration time at measurement T; atmosphere (N_2_, air, vacuum); confirm homogeneity (visual, DSC, density)	DES properties can be time-dependent, especially for high-viscosity systems; incomplete mixing produces heterogeneous samples
Temperature protocol	Essential	Measurement *T* reported; equilibration and heating/cooling rate often omitted	Report exact measurement *T* (±0.1 K); equilibration time at *T*; heating/cooling rate for DSC; specify whether ascending or descending *T* scan	DES transport properties are strongly *T*-dependent (VFT behavior); hysteresis effects can occur near Tg
Molar ratio precision	Strongly recommended	Nominal ratio reported (e.g., “1:2”); actual ratio rarely verified	Report weighed masses to ±0.1 mg; confirm stoichiometry by NMR integration or density comparison with reference data	Small deviations from eutectic ratio (±5 mol%) can shift viscosity by >30% and change phase behavior
H-bond definition in MD	Essential (computational)	Geometric criteria vary widely across publications (*D*–*A* distance 2.5–3.5 Å; angle 120–150°)	Report all geometric criteria (distance cutoff, angle cutoff, donor/acceptor atom types); perform sensitivity test showing variation of HB count with ±0.2 Å and ±10° changes; report both continuous and intermittent lifetimes	H-bond counts can vary by 20–40% depending on definition; comparability across studies requires transparent criteria
Force field and charge scheme	Essential (computational)	Force field named but often without version, charge source, or scaling	Report force field name and version; charge derivation method (RESP, AM1-BCC, etc.); charge scaling factor (if used); cite validation study; report whether 0.8 or 1.0 charge scaling is used for ions	Charge scaling (0.8 vs 1.0) can change computed viscosity by 2–5×; unscaled charges generally overestimate viscosity
Simulation details	Strongly recommended	Box size, trajectory length, and equilibration often incomplete	Report number of ion pairs and HBD molecules; box size (nm); total trajectory length and equilibrated portion; time step; thermostat/barostat; long-range electrostatics treatment	Finite-size effects and insufficient sampling are common; short trajectories (<5 ns) may not capture slow H-bond dynamics
Spectral assignment validation	Strongly recommended	Single-technique inference common (e.g., IR shift → “stronger H-bond”)	Cross-validate vibrational assignments with at least one independent method (NMR shifts, MD RDFs, DFT frequency calculations); report confidence level (strong/moderate/weak per triangulation criterion)	The same spectral shift can arise from multiple structural causes; overassignment of H-bond strength from IR alone is a persistent problem
Transport–structure correlation	Strongly recommended	Macroscopic η, σ, *D* reported; rarely connected to molecular H-bond descriptors	When transport data are reported alongside structural characterization, explicitly correlate to H-bond descriptors (lifetime, connectivity, motif population); report Walden plot position	Closing the structure–property gap is the central challenge; disconnected reporting of macroscopic and molecular data limits mechanistic insight
Interfacial effects	When applicable (electrochemistry, catalysis, extraction)	Neglected in most application studies; bulk properties assumed representative	When electrode, membrane, or surface processes are involved, discuss whether interfacial H-bond organization differs from bulk; perform interfacial MD or surface-sensitive experiments if feasible; at minimum, cite relevant interfacial literature	Interfacial H-bond topology controls electrode kinetics, wetting, and mass transfer; bulk-only characterization can seriously mislead mechanistic interpretation

**Table 5:** Quantitative Hydrogen-Bond and Transport Parameters across Representative DES Systems at 298 K^a^

DES system	Type	η (mPa·s)	σ (mS cm^–1^)	D_avg (10^–10^ m^2^ s^–1^)	Tg (K)	Tm or Teutectic (K)	Avg. HB per molecule	Cl^–^coord. no.	Dominant τ_HB (ps)	HBD self-assoc. (%)	**Dynamic heterogeneity (α**_**2**_,**max)**
Reline (ChCl:urea 1:2)	III	∼750	∼0.2	∼0.3	∼205	285	5.2	3.5–4.5	60–90	∼20	∼2.0
Ethaline (ChCl:EG 1:2)	III	∼36	∼7.6	∼2.5	∼170	207	4.0	3.0–4.0	25–40	∼35	∼1.0
Glyceline (ChCl:glycerol 1:2)	III	∼376	∼1.1	∼0.6	∼190	233	5.8	3.0–3.8	45–65	∼55	∼1.6
ChCl:malonic acid (1:1)	III	∼721	∼0.6	∼0.4	∼195	283	4.5	3.0–3.5	50–70	∼30	∼1.5
ChCl:oxalic acid (1:1)	III	∼3500	∼0.1	∼0.1	∼200	307	4.0	2.5–3.5	70–100	∼25	∼2.2
NADES: ChCl:glucose (1:1 + H_2_O)	NADES	∼8000 (neat)	<0.05	<0.1	∼215	—	6.5	3.0–4.0	80–120	∼65	∼2.5
NADES: ChCl:citric acid (1:1)	NADES	∼5000	∼0.05	∼0.1	∼210	—	5.5	2.5–3.5	70–100	∼45	∼2.0
Thymol:menthol (1:1)	V	∼25	∼0.01	∼3.0	∼155	250	1.8	N/A	8–15	∼60	∼0.5
Hydrophobic: TBACl:DecA (1:2)	Hydrophobic	∼180	∼0.3	∼0.5	∼180	265	3.0	2.0–3.0	30–50	∼40	∼1.3
Reline + 10 wt% H_2_O	III (hyd.)	∼80	∼3.5	∼1.5	∼175	—	4.0	2.8–3.5	30–50	∼15	∼1.0
Reline + 30 wt% H_2_O	III (hyd.)	∼8	∼25	∼12	∼140	—	2.5	2.0–3.0	10–20	∼5	∼0.3

a All values are at or near 298 K and atmospheric pressure unless indicated otherwise. η = dynamic viscosity; σ = ionic conductivity; D_avg = average self-diffusion coefficient (species-averaged from DOSY or MD); Tg = glass transition temperature; Tm = melting/eutectic temperature; HB per molecule = average total hydrogen bonds per molecule from MD; Cl^–^ coord. no. = average number of HBD H atoms within the first solvation shell of Cl^–^ from MD; τ_HB = dominant hydrogen-bond lifetime from MD autocorrelation; HBD self-assoc. = percentage of total HBD hydrogen bonds that are donor–donor (not involving Cl^–^); α_2_,max = maximum of the non-Gaussian parameter from MD. Values are representative ranges synthesized from the cited literature; exact numbers depend on force field, H-bond definition, water content, and temperature protocol. Dashes (—) indicate data not available or not applicable.

At least four classes of hydrogen-bond motifs operate simultaneously in a typical Type III DES: (i) ionic Cl^–^···H–O/N bonds, (ii) donor–donor self-association (O–H···O, N–H···O=C), (iii) cation-mediated contacts (Ch^+^ OH···Cl^–^, C–H···Cl^–^), and (iv) water-competitive motifs that become significant even at trace moisture levels. Their relative populations and lifetimes shift continuously with composition, temperature, and interfacial confinement, producing the characteristic macroscopic signatures of DES behavior.

The present study introduces the concept of competitive hydrogen-bond partitioning as an organizing framework for understanding DES behavior. The DES network is viewed as a dynamic competition among motif classes for a finite pool of donor and acceptor sites; the balance of this competition, quantified through motif populations, persistence distributions, connectivity indices, and heterogeneity metrics, controls macroscopic properties. A second central theme is the cooperativity–mobility tradeoff: cooperative charge spreading at Cl^–^ simultaneously deepens eutectic depression and stiffens the hydrogen-bond network, explaining why DESs such as reline combine very low melting points with viscosities exceeding 700 mPa·s. The scope is focused on the physical chemistry of hydrogen bonding; application-oriented aspects are discussed only insofar as they illuminate hydrogen-bond mechanisms.

## 2 Methods and Analytical Framework

### 2.1 The Challenge of Multimodal Characterization

Hydrogen bonding in DESs spans an unusually broad range of interaction types, energies, and timescales. Ionic Cl^–^···H–X bonds contribute 20–55 kJ mol^–1^, while cation-mediated C–H···Cl^–^ contacts may be as weak as 5–15 kJ mol^–1^;^13^ lifetimes range from subpicosecond librational fluctuations captured only by AIMD^22^ to persistent nanosecond-scale motifs detectable via dielectric spectroscopy.^17^ No single experimental or computational method can simultaneously resolve the energetics, geometry, dynamics, and spatial heterogeneity of such a network. Vibrational spectroscopy (FTIR, Raman) provides direct sensitivity to hydrogen-bond strength via O–H and N–H stretching frequency shifts but yields only time-averaged, spatially unresolved information;^25^ NMR offers composition-resolved chemical-shift perturbations and species-specific diffusion coefficients through DOSY but is limited by exchange averaging in viscous media;^26^ neutron diffraction with isotopic substitution and EPSR modeling delivers the most direct structural constraints—pair correlation functions *g*(*r*) and spatial density functions—but requires facility access and is not a routine laboratory technique.^14,27^ On the computational side, classical molecular dynamics (MD) provides nanosecond trajectories with full access to hydrogen-bond geometry, lifetimes, and cluster statistics, yet the results are critically dependent on the force field employed—charge scaling (0.8 vs 1.0), in particular, can alter computed viscosities by a factor of 2–5 and hydrogen-bond lifetimes by 20–40%.^28,29^ AIMD and DFT-MD resolve charge transfer and cooperativity with high fidelity but are limited to ∼100–300 atoms and trajectories of 10–100 ps, precluding the sampling of slow collective rearrangements.^22,30^

This complementarity of strengths and weaknesses motivates a systematic framework for combining techniques. Table 1 (see Section 3) compiles 12 methods—FTIR/ATR-IR, Raman/UVRR, ^1^H/^35^Cl NMR, DOSY NMR, neutron/X-ray scattering with EPSR, dielectric spectroscopy, DSC/TGA, sum frequency generation (SFG), classical MD, AIMD/DFT-MD, DFT cluster calculations, and machine-learning potentials—organized by category, accessible time scale/length scale, static versus dynamic capability, DES-specific pitfalls, and recommended method pairings. The corresponding information-content matrix (Figure 3) provides semiquantitative, expert-assessed scores for each method on a 1–5 scale across seven hydrogen-bond properties: strength and energy, topology and connectivity, dynamics and lifetimes, microheterogeneity, water competition, transport link, and interfacial structure (scoring rubric in Figure 3). Inspection of this matrix leads to a key conclusion: classical MD and AIMD score highest across the most properties (average scores of 4.3 and 4.4, respectively), but neither alone covers microheterogeneity, interfacial structure, and experimental validation simultaneously. Spectroscopic methods excel at probing hydrogen-bond strength and water competition but have limited access to network topology and dynamics. The highest-scoring single experiment is neutron/X-ray scattering (average 3.3), owing to its unique ability to constrain pair distances with subångström resolution, yet it provides no dynamic information.

### 2.2 The Triangulation Criterion

On the basis of these observations, the present study proposes a *triangulation criterion* for hydrogen-bond characterization in DES: any hydrogen-bond assignment or structural claim should be supported by evidence from at least two independent method categories—one providing structural or energetic constraints (spectroscopy, scattering, DFT) and one providing dynamic or statistical information (MD, DOSY, dielectric). This criterion formalizes what has been implicitly recognized in the best recent studies: Fetisov et al. validated AIMD-derived hydrogen-bond populations against experimental neutron scattering for reline;^22^ Zahn and coworkers combined machine-learning potentials with AIMD training data and then benchmarked against experimental density and viscosity;^18^ and Pandian et al. combined systematic FTIR, ^35^Cl NMR, and MD to track chain-length effects on Cl^–^ coordination across a homologous diol series.^24^ By contrast, single-technique inferences remain common in the literature—particularly the widespread assignment of “stronger” or “weaker” hydrogen bonds from IR band shifts alone, without independent structural validation. The sensitivity analysis in Table 4 (Section 3.8) shows that the same spectral shift can arise from composition-driven changes in the dielectric environment, donor self-association equilibria, or competitive hydration, underscoring the risk of overinterpretation.

A practical implication of the triangulation criterion is that computational studies should always include at least one experimental observable as a benchmark. For classical MD, force-field validation against both structural (radial distribution functions, coordination numbers) and dynamic (viscosity, diffusion coefficients, ionic conductivity) observables is essential; Table 6 compiles the validation status of eight force-field families used in DES simulations and identifies common pitfalls (Section 3.2). For AIMD, the limited trajectory length demands complementary classical MD or experimental lifetime data to contextualize short-time correlations. For spectroscopic studies, DFT frequency calculations on representative cluster models provide the most rigorous route to mode assignment in DES, where band overlap and multiple donor environments complicate deconvolution.^25,31^

**Table 6:** Classical Force Fields Commonly Used in DES Molecular Dynamics Simulations: Parameterization, Validation, and Limitations

Force field	Charge scheme	Charge scaling^a^	DES systems validated	H-bond properties reproduced	Known limitations/pitfalls	Critical methodological dependencies^b^
OPLS-AA (standard)	Fixed, 1.14*CM1A or RESP	None (*q* = ±1.0)	Reline, ethaline, glyceline	Structural (RDFs, coordination); qualitative H-bond trends	Severely overestimates viscosity (3–10×); dynamics too slow; ion pairing overestimated; poor transport properties	LJ ε/σ from OPLS94/2005 literature; electrostatics cutoff scheme (PME vs reaction field); box size ≥5 nm for transport
OPLS-DES (Doherty)	Fixed, RESP-derived	0.8 on ions	Reline, ethaline, glyceline, ChCl:levulinic acid	Viscosity, density, diffusion within ∼20–50% of experiment; improved dynamics vs unscaled	Charge scaling is empirical; may underestimate ion pairing; transferability to non-ChCl systems not guaranteed; HBD charges not scaled	Scaling factor 0.8 applied to ions only—changing to 0.7 or 0.9 shifts viscosity ≤2×; trajectory ≥10 ns required for converged viscosity; HB geometric cutoff sensitivity ±0.2 Å
GAFF + RESP	Fixed, RESP (HF/6–31G*)	0.8 on ChCl ions	Reline, ethaline, glyceline, aqueous DES	Density (∼1%); thermal conductivity; structural features; H-bond populations	Viscosity still overestimated by ∼50–100% for neat DES; charge scaling improves but does not fully resolve dynamics; HB lifetimes may be too long	RESP fitting basis set (HF/6–31G* standard; MP2/cc-pVTZ gives different charges); GAFF LJ parameters for O/N atoms; charge scaling applied to ions only; conformational sampling in RESP fitting
Refined ChCl:EG (Maginn group)	Fixed, scaled	σ-scaling + charge scaling	Ethaline (ChCl:EG) at multiple compositions and temperatures	Density, viscosity, ionic conductivity, diffusion coefficients quantitatively (∼10–20%)	System-specific; transferability to other HBDs requires reparameterization; additional scaling factors needed per HBD	σ-scaling factors derived specifically for EG hydroxyl groups; additional LJ rescaling per atom type; full reparameterization mandatory for each new HBD; composition-dependent validation essential
CGenFF/CHARMM-based	Fixed, CGenFF charges	Partial (0.8–0.9 on ions)	Reline, ethaline	Structure and short-time dynamics	Limited validation for DES; fewer benchmark studies than OPLS/GAFF; viscosity generally overestimated	CGenFF penalty scores must be checked (≤10 acceptable); torsion parameters for flexible HBD backbones; CMAP corrections for peptide-like donors; partial charge validation vs QM ESP
Polarizable FF (Drude oscillator)	Polarizable	N/A (self-consistent)	Reline (limited studies)	Improved charge delocalization; better capture of Cl^–^···HBD cooperativity	Very limited DES benchmarks; 3–10× computational cost; parametrization complex; few validated DES models available	Drude spring constant kD and atomic polarizabilities α must be fitted to QM polarizability data; Thole damping parameter strongly affects short-range electrostatics; dual Nosé–Hoover thermostat required for Drude degrees of freedom; SCF convergence threshold ≤ 10^–10^ kJ mol^–1^
AIMD-derived/reparameterized	DFT-derived (NBO, QTAIM)	System-specific	Reline, N-methylurea:ChCl	High-fidelity local H-bond energies; accurate charge redistribution	Not “force fields” per se; limited to training set; validation against macroscopic properties often not performed	DFT functional (PBE, BLYP, revPBE); dispersion correction (D3, D3BJ, DFTD4—lifetimes can differ 20–40%); basis set (DZVP-MOLOPT vs TZV2P); AIMD trajectory length for charge sampling (≥50 ps); cell size ≥64 molecules; NBO vs QTAIM partitioning scheme
Machine learning potentials (NNP, GAP, MACE)	Learned from AIMD	Implicit (training data)	Reline (proof-of-concept studies)	Near-AIMD accuracy for structure; promising for dynamics	Still in early development for DES; requires large AIMD training sets; limited transferability; overfitting risk; no standardized DES training protocols	AIMD reference level (functional + dispersion correction) determines accuracy ceiling; training set size (≥10^4^ configurations for DES); energy RMSE < 1 meV atom^–1^ and force RMSE < 50 meV Å^–1^ recommended; active learning loop for coverage of rare configurations; transferability across compositions not guaranteed

a “Charge scaling” refers to the practice of uniformly reducing the formal charges on ionic species (typically by 0.8×) to implicitly account for electronic polarization and charge transfer effects in fixed-charge simulations. This has become de facto standard for DES MD but introduces systematic approximations. Viscosity and diffusion are the most sensitive properties to force-field choice. For predictive DES design, force-field validation against both structural (RDF, coordination numbers) and dynamic (viscosity, diffusion, conductivity) observables is essential. Researchers are strongly encouraged to report the force field version, charge scaling factor, and validation metrics alongside any DES simulation results.

b Critical methodological dependencies are the key parameter choices and implementation decisions that most strongly affect the accuracy of the listed force-field approach for DES hydrogen-bond characterization. Changing these parameters outside the recommended ranges can degrade agreement with experiment by factors of 2–10 or more. They should be explicitly reported in any DES simulation study (see Table 4, reporting standards).

### 2.3 Hydrogen-Bond Descriptor Set

A second pillar of the analytical framework developed in this study is a set of six hydrogen-bond descriptors proposed for standardized DES characterization and predictive design (Table 3). These descriptors are ranked by priority and classified by current maturity:

(1) *HB motif population*—the fractional distribution of Cl^–^···H–O, Cl^–^···H–N, donor–donor, cation-mediated, and water-mediated motifs—is the most fundamental descriptor, directly capturing the local chemistry. It is obtainable by MD/AIMD through radial and angular distribution analysis, and experimentally through spectral deconvolution (IR, Raman) or ^35^Cl NMR line shape analysis.^26,32^ It is classified as routine by computation and moderate by experiment.
(2) *HB persistence distribution*—the survival probability *C*(*t*) or lifetime spectrum of each motif class, characterized by a mean lifetime τ_HB and stretching exponent β—separates strong–static from labile networks. This descriptor directly correlates with viscosity and diffusion more faithfully than average hydrogen-bond counts.^28,33^ Computationally routine, it remains experimentally emerging (accessible via NMR relaxation proxies and dielectric spectroscopy).
(3) *Network connectivity index*—a graph-theory metric encompassing average degree, clustering coefficient, and percolation threshold of the instantaneous hydrogen-bond network—captures topology beyond pairwise counts. Percolation analysis on MD trajectories distinguishes gel-like (percolating) from fluid (subpercolating) network states and has been shown to correlate strongly with the viscosity of ChCl-based DES across compositions.^34,35^ This descriptor is classified as emerging.
(4) *Competitive hydration index*—the fractional shift in motif populations (Δ_ionic, Δ_donor–donor) upon water addition from 0 to 10 wt%—quantifies moisture sensitivity. Hammond, Bowron, and Edler demonstrated by neutron diffraction that even modest hydration levels significantly restructure the DES network, with water preferentially inserting into Cl^–^ solvation shells and displacing HBD molecules.^36^ For hydrophobic DES, the structural disruption threshold is dramatically lower (1–3 wt%), making this descriptor essential for process robustness assessment.^37^
(5) *Dynamic heterogeneity metric*—the maximum of the non-Gaussian parameter α_2_(*t*), the ratio of fast to slow diffusion components, or the distribution width of local relaxation times—captures the spatial variation in molecular mobility that is a defining feature of DES dynamics. Spittle et al. showed that α_2_,max values for choline chloride–based systems range from ∼0.5 (dilute, homogeneous) to ∼2.5 (neat NADES, strongly heterogeneous), correlating directly with the departure from Stokes–Einstein behavior.^17^ This descriptor is routine by MD but experimentally early stage.
(6) *Interfacial HB partitioning*—the difference in motif populations and orientation order parameters between bulk (>3 nm from surface) and interfacial (<1 nm) regions—is the least mature but potentially most consequential descriptor for applied DES science. Hammond et al. demonstrated by neutron reflectometry and MD that the DES–electrode interface features Cl^–^ enrichment ∼2× the bulk density in the inner Helmholtz layer, with concomitant HBD depletion of ∼40%.^38^ This restructuring governs electrochemical double-layer capacitance, electrode kinetics, and catalytic selectivity, yet it remains grossly underreported.

These six descriptors span the full range from molecular (motif population) to mesoscale (network connectivity, heterogeneity) to interfacial (partitioning), and from static (populations) to dynamic (persistence, heterogeneity). The descriptor set is designed to be method-agnostic—each can in principle be estimated from either computation or experiment, or ideally both—and to be directly correlatable with macroscopic observables (Section 3.4, Figure 6).

### 2.4 Reporting Standards and Reproducibility

A persistent obstacle to systematic analysis across the DES literature is the lack of standardized reporting. Table 4 presents 10 minimum reporting requirements, organized by criticality level (essential, strongly recommended, when applicable). The most impactful is water content: Karl Fischer titration or thermogravimetric analysis should be performed before, during, and after measurement, because even 0.5 wt% H_2_O can shift viscosity by 10–20% and alter Cl^–^ coordination numbers measurably.^36,39^ For computational studies, the hydrogen-bond geometric definition (distance cutoff, angle cutoff, donor/acceptor atom types) and its sensitivity—typically tested by varying the cutoff by ±0.2 Å and ±10°—must be reported, since hydrogen-bond counts can vary by 20–40% depending on the criteria chosen.^28,40^ Force-field provenance, including name, version, charge derivation method, and scaling factor, is essential for reproducibility; unscaled charges generally overestimate viscosity by factors of 3–10.^29^ The adoption of these standards across the community would substantially improve cross-study comparability and accelerate the development of data-driven models for DES design.

All numerical ranges cited in this work are systematically documented with individual source data in Table S1 (Supporting Information).

## 3 Results and Discussion

### 3.1 The Hydrogen-Bond Landscape beyond HBA/HBD Binary Models

The conventional description of DES formation treats the system as a binary interaction between a hydrogen-bond acceptor (HBA, typically a quaternary ammonium halide) and a hydrogen-bond donor (HBD), forming a complexed anion of the type [Cl­(HBD)_*x*_]^−^ that reduces the lattice energy of the salt and frustrates crystallization.^1,2^ This picture correctly identifies chloride–HBD interactions as necessary for eutectic formation, but it obscures the competitive complexity that defines the liquid state. The hydrogen-bond landscape in a typical Type III DES is more accurately described as a four-component competition, illustrated schematically in Figure 1.

Figure 1 presents the multiscale hydrogen-bond organization of DESs across three spatial regimes. At the molecular scale (left panel), four classes of local motifs are identified: ionic hydrogen bonds (Cl^–^···H–O, Cl^–^···H–N), neutral donor–donor associations (O–H···O, N–H···O=C), cation-mediated contacts (Ch^+^ OH···Cl^–^, C–H···Cl^–^), and water-mediated interactions (H_2_O···Cl^–^, H_2_O···HBD). These motifs are not independent; they are connected by competition for a finite number of donor and acceptor sites and by cooperation through charge transfer and polarization. The essential insight of the competitive-partitioning framework is that these four motif classes draw on overlapping pools of donors and acceptors: a urea N–H group that forms a Cl^–^···H–N bond is unavailable for N–H···O=C self-association, and a Cl^–^ coordination site occupied by water is no longer accessible to the HBD. The equilibrium distribution of motifs is thus a zero-sum competition, modulated by temperature, composition, and the geometric constraints imposed by molecular architecture.

The center panel of Figure 1 illustrates how these local motifs generate mesoscale features: HBD-rich and ion-rich nanodomains, water channels, and dynamic heterogeneity with characteristic lifetimes spanning picoseconds to nanoseconds and spatial scales of 1–5 nm. The existence of such nanoscale segregation has been confirmed by neutron diffraction,^14^ small-angle X-ray scattering,^45^ and molecular dynamics simulations.^17,34^ The right panel of Figure 1 depicts the interfacial regime at an electrode or solid surface, where the hydrogen-bond network restructures into layered configurations that differ profoundly from the bulk, as discussed in Section 3.7. The bottom panel connects the network architecture to the macroscopic properties it governs: viscosity (η), ionic conductivity (σ), self-diffusion (*D*), glass transition temperature (Tg), melting temperature (Tm), selectivity, and interfacial reactivity. Each property is linked to a specific aspect of the hydrogen-bond network—viscosity to network persistence, conductivity to ion mobility versus correlation, diffusion to exchange kinetics, Tg/Tm to frustration and cooperativity—reinforcing the principle that structure–property relationships in DES are mediated through the hydrogen-bond competition.

The classification of DES types on the basis of their hydrogen-bond character is presented in Figure 2. This figure organizes the major DES classes—Types I through V, NADES, hydrophilic, and hydrophobic—by their dominant hydrogen-bond motifs and a qualitative indicator of hydrogen-bond contribution to eutectic formation. Type III systems (ionic, organic salt + HBD) exhibit the strongest hydrogen-bond involvement through Cl^–^···H–O/N ionic interactions combined with donor–donor self-association. The strength of these interactions is well documented: DFT cluster calculations by Ashworth et al. revealed nine distinct hydrogen-bond types in reline, with doubly ionic motifs (Ch^+^–OH···Cl^–^) exhibiting covalency comparable to strong neutral hydrogen bonds.^13^ Type V systems (molecular, nonionic HBD + HBA) rely on a fundamentally different bonding paradigm: neutral O–H···O hydrogen bonds operate synergistically with π–π stacking and London dispersion, producing eutectic depression without any ionic hydrogen-bond component.^21,46^ NADES feature multifunctional donors (polyols, sugars, organic acids) with multiple hydroxyl or carboxyl groups that create densely connected, multisite hydrogen-bond networks.^4^ The hydrogen-bond strength continuum at the bottom of Figure 2—from dispersion-dominated (Type V) to doubly ionic with charge transfer (Type III)—provides a first-order organizing principle, but it is important to emphasize that strength alone does not predict DES behavior; the topology, persistence, and cooperativity of the network are equally consequential.

The quantitative energy landscape is mapped in Figure 4. Panel (a) compiles interaction energy ranges from the computational literature for each motif class: ionic Cl^–^···H–N bonds span 25–55 kJ mol^–1^, Cl^–^···H–O bonds 20–50 kJ mol^–1^, doubly ionic Ch^+^–OH···Cl^–^ contacts 30–65 kJ mol^–1^, neutral donor self-association (O–H···O) 15–30 kJ mol^–1^, urea chains (N–H···O=C) 18–35 kJ mol^–1^, weak C–H···Cl^–^ contributions 5–15 kJ mol^–1^, water-competitive O–H···OH_2_ motifs 12–28 kJ mol^–1^, and non-hydrogen-bond cooperative interactions (π–π stacking 8–25 kJ mol^–1^, dispersion 2–10 kJ mol^–1^).^13,30,31,41^ The breadth of these ranges reflects the sensitivity of hydrogen-bond energetics to the local electrostatic environment, donor geometry, and cooperative context—a single Cl^–^···H–N bond in an isolated Cl^–^–urea dimer is substantially weaker than the same interaction type embedded in the full solvation shell of a cooperative DES. Panel (b) partitions the estimated energy contributions by motif type across six representative systems. In reline, ionic hydrogen bonds account for approximately 40% of the total cohesive interaction energy, followed by donor–donor self-association (20%), cation-mediated contacts (15%), and dispersion/other (10%). In Type V thymol:menthol, dispersion and π–π interactions dominate (∼40%), with neutral hydrogen bonds contributing ∼30% and ionic contacts entirely absent. In NADES (ChCl:glucose), the picture is further complicated by extensive intramolecular hydrogen bonding within the sugar donor, which competes with intermolecular coordination of Cl^–^. These fingerprints underscore the central argument: the balance of the hydrogen-bond competition, not the strength of any single motif, determines DES behavior.

Table 2 compiles the composition, dominant hydrogen-bond motifs, and physicochemical properties for twelve representative DES systems spanning all major categories. Several systematic trends emerge from the inspection of these data. First, viscosity spans 3 orders of magnitude (from ∼25 mPa·s for Type V thymol:menthol to >8000 mPa·s for neat ChCl:glucose NADES) and does not correlate with any single hydrogen-bond metric. Instead, it tracks with a combination of network connectivity and hydrogen-bond persistence: reline (η ≈ 750 mPa·s) and NADES systems exhibit high Cl^–^ coordination numbers (3.5–4.5) and long dominant τ_HB values (60–120 ps), while ethaline (η ≈ 36 mPa·s) and Type V systems show lower coordination numbers and shorter lifetimes. Second, the water sensitivity column reveals a critical design variable: hydrophobic DES undergo structural disruption at 1–3 wt% H_2_O, while hydrophilic ChCl-based systems tolerate 5–15 wt% before major network reorganization occurs. Third, Tg values cluster into two regimes—high-connectivity ionic systems (Tg ≈ 190–215 K) and low-connectivity molecular systems (Tg ≈ 145–170 K)—suggesting that glass formation in DES is governed by the percolation threshold of the hydrogen-bond network rather than by molecular weight or cohesive energy density alone.

Types I, II, and IV DES, which employ metal halides (e.g., FeCl_3_, SnCl_2_, ZnCl_2_) as hydrogen-bond acceptors, fall outside the scope of the present study. Systematic liquid-phase hydrogen-bond characterization data for these systems remain sparse compared with the extensive Type III literature, and the molecular picture is complicated by two features absent in organic-salt-only DES: (i) the metal cation acts simultaneously as a Lewis-acidic coordination center and an electrostatic sink, so that Zn^2+^–O/N dative bonds compete directly with Cl^–^···H–X hydrogen bonds for HBD coordination; and (ii) the halometallate anion speciation—e.g., [ZnCl_3_(HBD)]^−^, [ZnCl_4_]^2–^, [Sn_2_Cl_5_]^−^—is itself a composition- and temperature-dependent equilibrium, adding a speciation dimension to the competition that is absent in the binary Cl^–^ /HBD framework.^47,48^

Notwithstanding these additional complexities, several elements of the competitive-partitioning framework developed here are in principle transferable. Motif population and network connectivity descriptors can be extended to include metal–ligand coordination alongside Cl^–^···H–X bonds; the triangulation criterion requiring evidence from at least two independent method categories applies without modification; and the cooperativity–mobility tradeoff may manifest through Lewis-acid-enhanced charge delocalization across the metal coordination shell, an analogue of the chloride charge-spreading mechanism central to Type III systems. Recent DFT/QTAIM studies of ChCl:SnCl_2_ and ChCl:ZnCl_2_ confirm the coexistence of traditional hydrogen bonds and electrostatic interactions around the chlorometallate anion,^47^ and periodic DFT analysis of the ZnCl_2_(urea)_2_ crystal reveals that ZnCl_2_ acts as a structural disruptor of the urea hydrogen-bond network in a manner qualitatively analogous to the charge-spreading role of Cl^–^ in reline.^48^ Extending the full descriptor set—including HB persistence distributions, dynamic heterogeneity, and interfacial partitioning—to Types I/II/IV DES represents a priority for future work.

### 3.2 Experimental and Computational Characterization

The information-content matrix (Figure 3) provides a semiquantitative, expert-assessed basis for selecting method combinations. The scores reflect comparative literature assessment and expert consensus rather than directly measured performance thresholds; users should treat the matrix as a structured guide rather than an objective ranking. Twelve methods are scored across seven hydrogen-bond properties on a 1–5 scale: HB strength and energy, HB topology and connectivity, HB dynamics and lifetimes, microheterogeneity, water competition, transport link, and interfacial structure. The matrix reveals several instructive patterns that merit careful analysis.

Classical MD and AIMD/DFT-MD achieve the highest cumulative scores (30 and 30 out of 35, respectively), reflecting their unmatched ability to simultaneously access hydrogen-bond geometry, dynamics, topology, and composition dependence. However, these high aggregate scores mask important complementarities. Classical MD excels at dynamics and lifetimes (score of 5) and microheterogeneity (score of 5) because it can access nanosecond trajectories and large simulation cells (>10 nm), but it receives a lower score on HB strength and energy (3) because force-field-dependent charge distributions cannot capture the quantum-mechanical subtleties of charge transfer and cooperativity. AIMD reverses this pattern: it scores 5 on HB strength (direct access to electronic structure) and 5 on HB dynamics (no empirical HB definition needed), but only 4 on microheterogeneity and topology because small cell sizes (100–300 atoms) and short trajectories (10–100 ps) preclude adequate statistical sampling of slow collective phenomena.^22,30^ It must be emphasized, however, that these high information-content scores presuppose careful method selection and rigorous experimental validation—conditions that are frequently not met in practice. AIMD results in particular are sensitive to the choice of exchange-correlation functional (e.g., PBE vs BLYP vs revPBE), dispersion correction scheme (D3, D3BJ, or DFTD4), basis set completeness, and simulation cell size; different functional/dispersion combinations applied to the same DES can yield hydrogen-bond lifetimes and interaction energy partitions that differ by 20–40%, a range comparable to the differences between DES types themselves. Classical MD is correspondingly constrained by force-field parametrization quality, charge-scaling choices, and the geometric definition of the hydrogen bond (Table 6). The sensitivity of computed hydrogen-bond populations to the distance and angle cutoffs alone can reach 20–40% (Table 4, Section 2.4). In practice, computational methods reach the information-content scores tabulated in Figure 3 only when their outputs have been validated against at least one independent experimental observable—a requirement formalized in the triangulation criterion (Section 2.2) and discussed in detail in the force-field benchmark assessment (Table 6). Readers should therefore treat the matrix scores (Table 7) as aspirational upper bounds for well-validated studies, not as properties of the computational methods themselves.

**Table 7:** 

Score	Definition
1	The technique provides no direct or useful information on this hydrogen-bond property, or the information is entirely indirect and requires assumptions that introduce unacceptable ambiguity (e.g., DOSY NMR on HB topology).
2	Low-resolution or highly indirect information; the technique is sensitive to this property but quantitative extraction requires significant assumptions, model dependence, or complementary data (e.g., FTIR on microheterogeneity via band broadening).
3	Moderate, ensemble-averaged information; the technique accesses the property in a meaningful but incomplete way—for example, with no spatial resolution, no time resolution, or restricted to a subset of the relevant length/time scales (e.g., neutron scattering on HB dynamics).
4	High-quality information with specific, well-defined limitations such as restricted time scale, requirement for facility access, need for complementary modeling for full interpretation, or modest statistical sampling (e.g., AIMD on microheterogeneity).
5	State-of-the-art, comprehensive information; the technique provides direct, quantitative, and well-validated access to this property with no major intrinsic limitation for the DES context (e.g., classical MD on HB dynamics and lifetimes; DOSY NMR on transport link).

Among experimental methods, neutron/X-ray scattering with EPSR achieves the highest cumulative score (19 out of 35), owing to its unique capacity to resolve pair distances with subångström precision and provide three-dimensional spatial density functions.^14,27^ Yet it scores only 3 on dynamics and 3 on interfacial structure, because it provides ensemble-averaged snapshots with no time resolution and requires specifically designed interfacial geometries for reflectometry experiments. Vibrational spectroscopy (FTIR, Raman) provides excellent sensitivity to hydrogen-bond strength (scores of 4) and offers in situ temperature-scan capability, but it scores only 1–2 on microheterogeneity because it reports spatially averaged spectra that cannot distinguish coexisting nanoscale domains.^16,25^ DOSY NMR stands out for its unique combination of transport sensitivity (score of 5) and species-resolved mobility (score of 5), enabling direct determination of self-diffusion coefficients for each DES component independently,^26^ but it cannot directly probe hydrogen-bond geometry (score of 1). Dielectric spectroscopy provides an exceptionally broad dynamic range (picoseconds to microseconds in the frequency domain) and connects relaxation processes to ionic transport (scores of 4 on dynamics and 5 on transport), but deconvolution of overlapping relaxation modes is complex and model-dependent.^17^

The force-field landscape for DES simulation is assessed in Table 6, which evaluates eight force-field families—standard OPLS-AA, OPLS-DES (Doherty),^28^ GAFF+RESP, the refined ethaline model of Ferreira et al.,^29^ CGenFF/CHARMM-based, polarizable Drude-oscillator models, AIMD-derived reparameterizations, and machine-learning potentials^18^ against criteria including charge scheme, charge scaling, validated DES systems, reproduced hydrogen-bond properties, and known limitations. The dominant finding is that charge scaling has become de facto standard for fixed-charge DES simulations. Reducing ionic charges by 0.8× implicitly accounts for electronic polarization and charge transfer that would otherwise require explicit many-body treatments; this single empirical correction improves computed viscosities from 3–10× overestimation (unscaled) to 20–50% agreement with experiment.^28,29^ However, charge scaling introduces systematic biases: it underestimates ion pairing and may distort the relative populations of ionic versus donor–donor motifs. The limitation is particularly relevant for the competitive-partitioning framework, where accurate relative motif populations are essential.

Machine-learning potentials represent the most promising frontier. Shayestehpour and Zahn trained neural-network potentials on AIMD reference data for reline and demonstrated near-DFT accuracy for liquid structure at classical-MD computational cost.^18^ The approach implicitly captures charge transfer, polarization, and cooperativity without empirical scaling. However, three limitations currently constrain its deployment for DES design: (i) extensive AIMD training data are required for each new composition, limiting transferability across the DES chemical space; (ii) validation against macroscopic transport properties (viscosity, diffusion, conductivity) remains sparse; and (iii) no standardized training protocols for DES force fields have been established. Nevertheless, the trajectory from fixed-charge models (2014–2018) through scaled models (2016–present) to machine-learning potentials (2023–present) points clearly toward a future in which DES hydrogen-bond simulations achieve the accuracy currently reserved for small-molecule quantum chemistry.

### 3.3 Charge Spreading and Cooperativity

The electronic mechanism underlying DES stability extends beyond simple hydrogen-bond formation to involve cooperative charge redistribution across the anion solvation shell. This phenomenon—charge spreading—is analyzed in Figure 5 using a three-panel layout that traces the concept from molecular mechanism through energetic consequences to macroscopic design implications. Panel (a) contrasts the charge redistribution in two environments: an isolated pairwise Cl^–^···H–N­(urea) interaction (left) versus the cooperative, multiply coordinated Cl^–^ in a full solvation shell (right). In the isolated motif, charge transfer from the donor to chloride is modest (Δ*q* ≈ 0.03 e, NBO analysis,^41^ E_HB ≈ −35 kJ mol^–1^). This finding is consistent with the AIMD analysis of Zahn, Kirchner, and Mollenhauer,^41^ who showed that the negative charge transferred from Cl^–^ to urea in individual Cl^–^–urea pairs is surprisingly small, leading them to question whether charge delocalization alone is responsible for the depressed melting point. The resolution of this apparent paradox lies in the cooperative, many-body nature of the full solvation shell. When 3–5 HBD molecules simultaneously coordinate Cl^–^—as occurs in the liquid state (Table 2, Cl^–^ coordination numbers of 3.0–4.5)—cumulative charge transfer reaches ΣΔ*q* ≈ 0.13 e, NBO analysis,^13,41^ and the total interaction energy (ΣE_HB ≈ −160 kJ mol^–1^) exceeds the sum of isolated pairwise contributions by 15–40%, demonstrating positive cooperativity.^13^ The reduced effective charge on Cl^–^ (from approximately −0.85 e in the isolated ion to −0.72 e in the coordinated state (NBO-derived effective charges, ref^41^)) reflects exactly the charge spreading phenomenon: the key point is that the effect is negligible for a single donor but becomes substantial in the many-body context of the liquid. This explains why pairwise DFT calculations underestimate the charge-transfer contribution while AIMD simulations with full solvation shells recover it. It should be noted that partial atomic charges are not quantum-mechanical observables and are sensitive to the population analysis scheme employed. QTAIM and Hirshfeld analyses of the same Cl^–^–urea interactions yield smaller absolute charge-transfer values than NBO, while preserving the qualitative cooperative trend; CM5 and ESP-fitted charges fall between these bounds. The mechanistic conclusion—that cooperative multidonor coordination progressively reduces the effective negative charge on Cl^–^, thereby facilitating additional donor binding—is robust across all schemes. The absolute values reported here (0.03 e, 0.13 e, −0.85 e, −0.72 e) should therefore be regarded as order-of-magnitude estimates rather than precise quantities.

Critically, the cooperativity operates in both directions. As charge transfers from each HBD to Cl^–^, the partially neutralized anion becomes a better hydrogen-bond acceptor for subsequent donors because the electrostatic repulsion between the anion and the approaching donor lone pair is reduced. Simultaneously, the charge-depleted HBD becomes a slightly better donor for its next interaction (whether with another Cl^–^ or with a neighboring HBD). This bidirectional feedback creates a network-level stabilization that cannot be predicted from pairwise interaction energies alone and that fundamentally distinguishes the liquid DES from a simple mixture of its components.

Panel (b) of Figure 5 presents the energy diagram for sequential hydrogen-bond addition to Cl^–^, contrasting cooperative and anticooperative pathways. In the cooperative regime (teal curve, characteristic of reline-like systems), each additional donor contributes a progressively larger stabilization increment: −35 kJ mol^–1^ for the first donor, −42 kJ mol^–1^ for the second, −45 kJ mol^–1^ for the third (representative values estimated from DFT cluster calculations in refs 13,31,41 ), with the cumulative energy falling well below the additive reference (gray dashed line). In the anticooperative regime (magenta curve), steric crowding or electrostatic saturation causes diminishing returns: −28, −18, −12 kJ mol^–1^ for successive donors (representative estimates, refs 13,31,41 ). These incremental energies represent total interaction energy gains upon sequential donor addition to the Cl^–^ coordination shell and therefore include contributions from donor–donor electrostatic and steric interactions in addition to the Cl^–^···H–X bond itself. Rigorous separation of these components would require energy decomposition analysis (e.g., SAPT, ALMO-EDA, or Su–Li EDA), which was not uniformly applied in the cited cluster studies. The cooperative versus anticooperative distinction in Figure 5b therefore describes the net many-body stabilization trend—a qualitative descriptor that is physically meaningful and supported by the literature—rather than a quantitative hydrogen-bond-only energy partition. This limitation does not affect the mechanistic conclusions drawn from the cooperative–mobility tradeoff, which are based on the sign and direction of the cooperativity (positive vs negative) rather than on the absolute incremental values. The practical distinction is consequential: cooperative systems form deep eutectics with large ΔTm but inevitably high viscosities, because the same charge-spreading mechanism that destabilizes the crystal simultaneously creates persistent, rigid hydrogen-bond topologies in the liquid. Anticooperative systems exhibit weak eutectic depressions and, in some cases, fail to form DES altogether despite possessing strong pairwise hydrogen-bond donors. This framework explains why some HBA/HBD combinations with individually strong hydrogen bonds do not produce stable DES: pairwise strength is necessary but not sufficient; cooperative amplification within the many-body solvation shell is the additional requirement.

Panel (c) distills three principal consequences for DES behavior, presented as numbered boxes linked to a cooperativity–mobility design axis. The first consequence—melting-point depression—follows directly: cooperative charge transfer destabilizes the crystal lattice of the pure salt (Tm (ChCl) = 302 °C → Tm (reline) = 12 °C). The second consequence—the viscosity paradox—is the central insight: the same cooperative network that depresses Tm creates persistent, long-lived hydrogen-bond topologies (τ_HB = 60–90 ps for reline, Table 5), explaining the seemingly contradictory combination of very low melting points with viscosities of ∼750 mPa·s. The third consequence—DES formation criteria—states that successful eutectic formation requires not merely strong pairwise interactions but sufficient cooperative frustration among multiple competing motifs to prevent the system from finding a crystalline energy minimum.

The cooperativity–mobility tradeoff, presented as a gradient bar at the bottom of panel (c), defines a design axis with three regimes: low cooperativity (Type V, hydrophobic DES—weak eutectic depression, low viscosity, fast dynamics, limited solvation), moderate cooperativity (ethaline, hydrated DES—balanced properties, the design sweet spot for most applications), and high cooperativity (reline, NADES—deep eutectic, very high viscosity, slow dynamics, strong solvation but poor transport). The identification of this design axis—and its quantitative connection to charge-spreading parameters—represents a shift from empirical DES formulation toward rational, hydrogen-bond-informed design.

### 3.4 Structure–Property Correlations

The connection between hydrogen-bond network descriptors and macroscopic observables is quantified in Figure 6 through four cross-system correlations, supported by the comprehensive data in Table 5, which provides quantitative hydrogen-bond and transport parameters at 298 K for 11 representative DES systems. The data span 3 orders of magnitude in viscosity (8–8000+ mPa·s) (Table S1, Supporting Information), 2 orders of magnitude in conductivity (0.01–25 mS cm^–1^) (Table S1, entry 34), and nearly 2 orders of magnitude in self-diffusion (0.1–12 × 10^–10^ m^2^ s^–1^) (Table S1, Supporting Information).

Figure 6 panel (a) plots viscosity (logarithmic scale) against a normalized hydrogen-bond connectivity index. The correlation is approximately exponential: a doubling of the connectivity index (from ∼2.2 for hydrated DES at 30 wt% water to ∼4.0 for reline) produces roughly 2 orders of magnitude increase in viscosity. This exponential dependence is physically meaningful: in network-forming liquids, transport requires cooperative rearrangement of multiple bonds simultaneously, and the number of such rearrangements needed for macroscopic flow scales exponentially with network density above the percolation threshold.^34,45^ The key mechanistic insight is that viscosity tracks with network topology, not with hydrogen-bond strength per se: ethaline and reline have comparable ionic hydrogen-bond energies (Figure 4a) but differ dramatically in connectivity (ethaline ∼3.0 vs reline ∼3.8, Table 5), producing a 20-fold viscosity difference (36 vs 750 mPa·s).

Panel (b) presents ionic conductivity (logarithmic scale) versus average hydrogen-bond lifetime, revealing an inverse relationship. Longer hydrogen-bond lifetimes reduce the rate of ion exchange and cage-breaking events that enable ionic transport: reline (τ_HB ≈ 75 ps) (Table S1, entry 39) exhibits σ ≈ 0.2 mS cm^–1^, while hydrated DES at 30 wt% water (τ_HB ≈ 15 ps) (Table S1, entry 40) reaches σ ≈ 25 mS cm^–1^. The decay in conductivity with increasing lifetime is steeper than a simple inverse relationship, suggesting that collective network relaxation—the concerted breaking and reforming of multiple hydrogen bonds to enable ion displacement—is the rate-limiting process. This is consistent with the departure from Stokes–Einstein behavior observed in DES:^17,20^ if individual hydrogen-bond breaking were rate-limiting, viscosity and conductivity would be related by the simple Walden rule, whereas the observed deviations indicate that correlated multibond dynamics dominate transport.

Panel (c) presents self-diffusion coefficients versus connectivity index, showing exponential decay that mirrors the viscosity correlation through the Stokes–Einstein relation. The convergence of panels (a) and (c) toward the same exponential form is reassuring and confirms that the connectivity index is a robust descriptor of both rotational and translational mobility in DES. Panel (d) correlates glass transition temperature with average hydrogen-bond lifetime, revealing a positive linear trend (Tg increases by approximately 1 K per 1 ps increase in τ_HB). This linear relationship suggests that the energetic barrier to structural relaxation—which determines Tg through the Vogel–Fulcher–Tammann relationship—is set primarily by the mean hydrogen-bond lifetime rather than by the total interaction energy.

The hydrated DES data points (reline + 10 and reline + 30 wt% H_2_O) show that water shifts systems continuously along all four correlations; the detailed mechanism is analyzed in Section 3.5.^36^

Inspection of Table 5 reveals a critical multivariate insight: no single descriptor predicts all properties simultaneously. Viscosity correlates most strongly with network connectivity and τ_HB; conductivity with τ_HB and Cl^–^ coordination; Tg with connectivity and self-association fraction; and dynamic heterogeneity with the combination of connectivity, self-association, and τ_HB distribution width. This irreducible multivariate dependence justifies the six-descriptor framework of Table 3: reductive single-descriptor models necessarily fail to capture the competitive, multimotif character of DES hydrogen-bond networks.

### 3.5 Water as a Competitive Partner

Water is never a passive bystander in DES chemistry. Even trace moisture levels alter the hydrogen-bond competition by inserting water molecules into the Cl^–^ solvation shell, displacing HBD donors, and creating new H_2_O-mediated exchange pathways that accelerate dynamics. The hydration response of DES is remarkably nontrivial: water is simultaneously a network disruptor (breaking Cl^–^···HBD contacts) and a network mediator (forming H_2_O-bridged pathways between ions), and the balance between these roles shifts with concentration. This dual character is mapped in Figure 7 for two contrasting regimes.

Panel (a) presents the hydration regime map for hydrophilic DES (e.g., ChCl-based systems), showing four normalized response curves—DES hydrogen-bond network persistence (blue), ion pairing/clustering (orange), molecular mobility (green), and Cl^–^ coordination number (purple, right axis)—as a function of water content from 0 to 60 wt%. Four regimes are identified. In the trace-water regime (<3 wt%, [≈<31 mol% relative to the reline formula unit]), water molecules insert individually into the Cl^–^ first solvation shell with minimal perturbation to the overall network topology; this regime is experimentally significant because even “dried” DES samples typically retain 0.5–2 wt% (approximately 7–23 mol%) residual moisture, and as Table 4 emphasizes, the failure to report this water content is the single largest source of irreproducibility in the DES literature.^36,39^ In the competitive-hydration regime (3–15 wt%, [≈31–72 mol%]), H_2_O molecules progressively displace HBD molecules from Cl^–^ coordination. The consequences are measurable and correlated: viscosity decreases by up to 50%, ionic conductivity increases, and the Cl^–^ coordination environment shifts from HBD-dominated to mixed HBD/H_2_O. Critically, the hydrogen-bond network persistence declines faster than the ion pairing, indicating that the first structural casualty of hydration is the extended donor–donor and Cl^–^···HBD hydrogen-bond mesh rather than the electrostatic ion pair itself.

The network-reorganization regime (15–30 wt%, [≈72–86 mol%]) is qualitatively different: the hydrogen-bond topology undergoes wholesale restructuring. HBD–HBD and Cl^–^–HBD motifs are replaced by H_2_O···Cl^–^ and H_2_O···HBD interactions at a rate that exceeds simple dilution, because water is a stronger hydrogen-bond donor per unit volume than most organic HBDs. The percolating DES network fragments into isolated clusters embedded in a water-rich matrix, and the system begins to exhibit electrolyte-like behavior—Debye–Hückel screening, ion-pair dissociation, and Walden-rule-compliant transport.^36^ Above 30 wt% (approximately >86 mol%)—the aqueous-DES regime—the solution is better described as a concentrated aqueous electrolyte with dissolved organic components. The neutron diffraction work of Hammond, Bowron, and Edler provided the first direct structural evidence for this transition, showing that the DES nanostructure undergoes an unusual and continuous transformation rather than a sharp phase boundary.^36^

Panel (b) shows the dramatically different hydration response of hydrophobic DES (e.g., TBACl:decanoic acid), plotted over a much narrower water range (0–10 wt%, [approximately 0–79 mol%, relative to the TBACl:DecA formula unit]). Four response curves track hydrogen-bond network persistence (blue), acid dimer population (red), ion pair reorganization (orange), and phase separation tendency (gray). The structural disruption threshold is compressed: 1–3 wt% H_2_O­(approximately 26–52 mol%) is sufficient to break acid dimer motifs, reorganize ion pairing, and in some cases trigger macroscopic phase separation. This extreme sensitivity arises because hydrophobic DES rely on a sparse hydrogen-bond network reinforced predominantly by dispersion; water, being a far stronger hydrogen-bond donor than decanoic acid or menthol, catastrophically disrupts the balance by preferentially solvating any available ionic sites while remaining immiscible with the hydrophobic matrix.^37^ The practical implication for industrial process design is clear: hydrophobic DES applications require stringent anhydrous conditions that are difficult to maintain outside laboratory settings.

A note on the compositional scale used in this section is warranted. Hydration levels throughout this work are reported in wt% to maintain consistency with the experimental literature, in which water content is routinely determined by Karl Fischer titration and expressed on a mass basis. However, because the molar mass of a reline formula unit (ChCl·2urea, *M*_r_ ≈ 260 g mol^–1^) is approximately fourteen times that of water (18 g mol^–1^), the wt% and mol% scales diverge dramatically: 10 wt% H_2_O in reline corresponds to approximately 62 mol% water, and 30 wt% to approximately 86 mol%. At the latter composition, the solution is more accurately described as a dilute aqueous electrolyte containing dissolved choline, chloride, and urea than as a hydrated DES. This molar-fraction perspective reinforces the conclusion of Hammond, Bowron, and Edler^36^ that the structural transition from DES to aqueous electrolyte is continuous rather than abrupt, and that even modest wt% additions represent substantial mole-fraction perturbations of the DES network. For hydrophobic DES with still larger formula-unit molar masses (e.g., TBACl:DecA, *M*_r_ ≈ 622 g mol^–1^), the discrepancy is even more pronounced: 3 wt% H_2_O already corresponds to approximately 52 mol% water. Mol% equivalents for all wt% values cited in this section are provided inline above; Table 5 includes both scales for the hydrated reline entries. Researchers are encouraged to report water content in both units, as recommended in Tables 4 and 6.

The quantitative impact of hydration on the full property set is captured in Table 5 through the entries for reline, reline + 10 wt% H_2_O, and reline + 30 wt% H_2_O. At 10 wt% (approximately 62 mol% H_2_O relative to the reline formula unit), viscosity drops from ∼750 to ∼80 mPa·s (nearly 10-fold), conductivity increases from 0.2 to 3.5 mS cm^–1^, and the Cl^–^ coordination number decreases from 3.5–4.5 to 2.8–3.5 as water partially replaces HBD donors. The average number of hydrogen bonds per molecule decreases from 5.2 to 4.0, and the dynamic heterogeneity parameter α_2_,max drops from 2.0 to 1.0, indicating a substantially more homogeneous dynamic landscape. At 30 wt% (approximately 86 mol%), the transformation is effectively complete: η ≈ 8 mPa·s, σ ≈ 25 mS cm^–1^, D_avg ≈ 12 × 10^–10^ m^2^ s^–1^, and α_2_,max ≈ 0.3—values that are indistinguishable from those of concentrated aqueous electrolytes. The HBD self-association fraction collapses from ∼20% to ∼5%, confirming that the donor–donor network has been almost completely dismantled by water intrusion.

These hydration data have an important implication for the descriptor framework (Table 3, descriptor 4): the competitive hydration index—defined as the fractional shift in ionic motif population upon water addition from 0 to 10 wt%—is a direct, quantitative measure of process robustness. A DES with a small hydration index (e.g., ethaline, Δ_ionic ≈ −8%) can tolerate ambient moisture with modest property changes, while one with a large index (e.g., hydrophobic TBACl:DecA, Δ_ionic ≈ −60%) demands rigorous anhydrous handling. Incorporating this descriptor into predictive screening would allow the systematic elimination of candidates unsuitable for target operating environments.

### 3.6 Dynamic Heterogeneity and Microheterogeneity

One of the most consequential features of DES—and one that has received rapidly growing attention—is the coexistence of dynamically distinct molecular environments within a macroscopically homogeneous liquid. This dynamic heterogeneity arises directly from the competitive hydrogen-bond landscape: regions enriched in ionic motifs (high Cl^–^ density, strong and persistent hydrogen bonds) exhibit slow dynamics and high local viscosity, while HBD-rich or water-rich regions display faster relaxation and greater molecular mobility.^17,19,20^

Figure 8 presents five complementary views of this phenomenon. Panel (a) provides a schematic of the nanoscale domain structure, showing coexisting HBD-rich nanodomains (blue), ion-rich domains (red), and water clusters (purple) in a region near a solid interface. The domains are separated by hydrogen-bond exchange pathways (dashed arrows) that mediate mass and energy transfer between environments at length scales of 1–5 nm (Table S1, Supporting Information). This spatial organization has been confirmed by multiple independent approaches: neutron diffraction reveals pair-correlation features consistent with nanoscale segregation;^14^ MD simulations produce spatial density functions with alternating ion-rich and HBD-rich shells;^33,34^ and ultrafast optical spectroscopy detects multiple solvation environments with distinct relaxation timescales.^19^ The domain structure is not static: MD trajectories show that domains exchange content on timescales of 50–200 ps (Table S1, Supporting Information), faster than macroscopic diffusion but slower than individual hydrogen-bond breaking (∼5–20 ps) (Table S1, Supporting Information).

**Figure 8:**
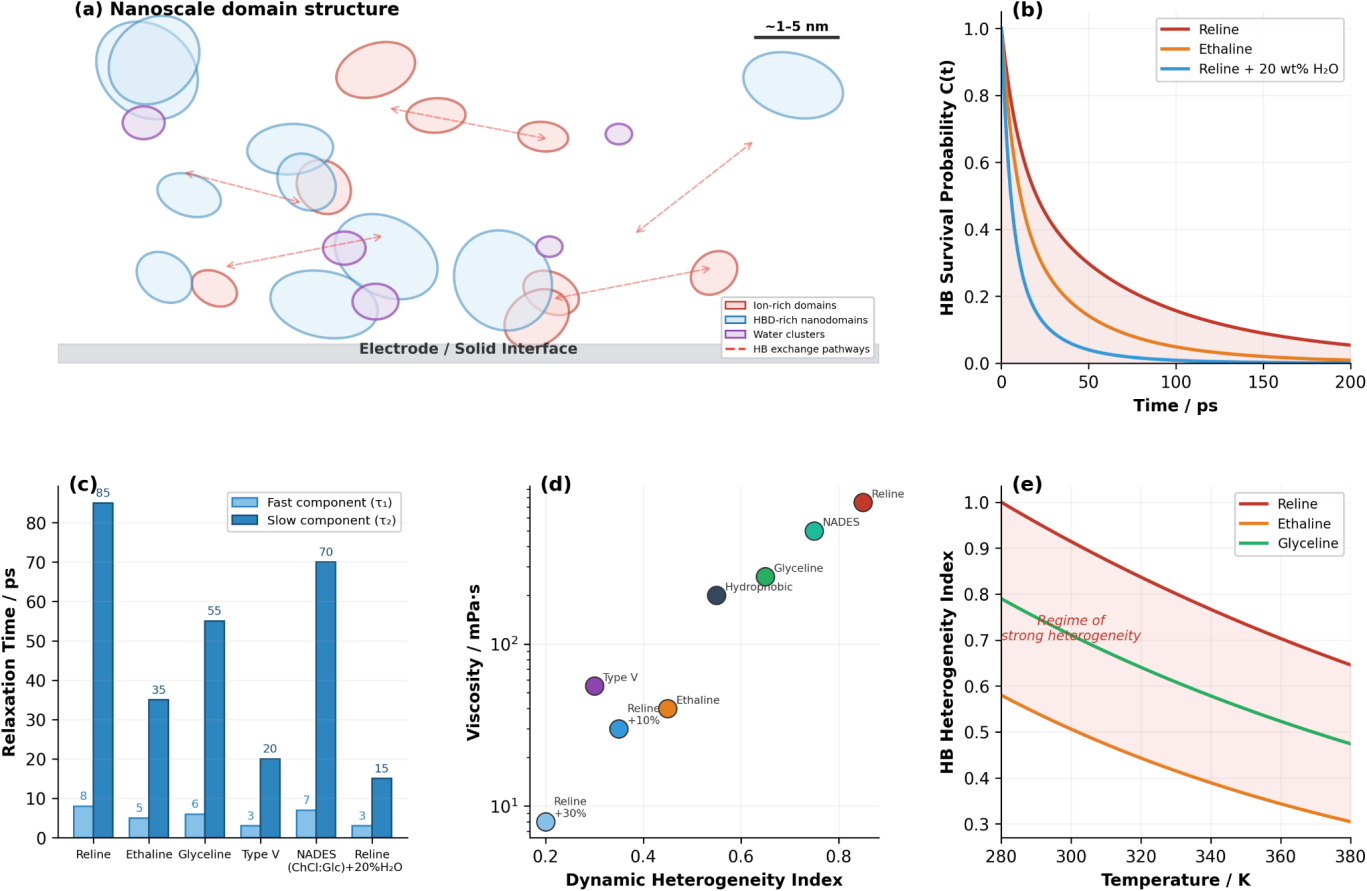
Dynamic heterogeneity and microheterogeneity in deep eutectic solvents. (a) Schematic representation of nanoscale domain structure showing coexisting HBD-rich nanodomains (blue), ion-rich domains (red), and water clusters (purple), with hydrogen-bond exchange pathways (dashed arrows) operating near a solid interface. Scale bar: ∼1–5 nm. (b) Hydrogen-bond survival probability *C*(*t*) for reline, ethaline, and hydrated reline (20 wt% H_2_O), illustrating the stretched-exponential decay characteristic of heterogeneous environments; reline shows the slowest decay consistent with its strong, persistent network. (c) Fast (τ_1_, light blue) and slow (τ_2_, dark blue) relaxation time components for representative DES systems, demonstrating the bimodal character of hydrogen-bond dynamics across DES types. (d) Viscosity versus dynamic heterogeneity index for selected systems, confirming that higher dynamic heterogeneity correlates with higher viscosity. Bubble size is proportional to H-bond connectivity. (e) Temperature dependence of the H-bond heterogeneity index for reline, ethaline, and glyceline, showing that reline maintains the strongest heterogeneity across the entire temperature range, while ethaline transitions toward more homogeneous dynamics above ∼340 K.

Panel (b) displays the hydrogen-bond survival probability *C*(*t*) for reline, ethaline, and hydrated reline (20 wt% H_2_O). All three systems exhibit stretched-exponential decay—*C*(*t*) ∼ exp­[−(*t*/τ)^β] with β < 1—the hallmark signature of heterogeneous dynamics. In a dynamically homogeneous liquid, *C*(*t*) would decay as a simple exponential (β = 1); the departure from this form reflects the superposition of fast and slow relaxation processes arising from molecules in different local environments.^33^ Reline exhibits the most strongly stretched decay (β ≈ 0.55, τ_HB ≈ 75 ps) (Table S1, Supporting Information), consistent with its strong, highly connected network; ethaline is less stretched (β ≈ 0.65, τ_HB ≈ 32 ps) (Table S1, Supporting Information); and hydrated reline approaches single-exponential behavior as water homogenizes the dynamic landscape. The stretching exponent β is itself a quantitative descriptor of heterogeneity: lower values indicate broader distributions of local relaxation environments and greater disparity between the fastest and slowest molecular populations.

Panel (c) decomposes the relaxation into fast (τ_1_, light blue) and slow (τ_2_, dark blue) components for five representative systems: reline, ethaline, glyceline, Type V (thymol:menthol), and NADES (ChCl:glucose). Reline exhibits the largest τ_2_/τ_1_ ratio (∼10) (Table S1, entry 58), meaning that its slow component—attributed to strongly coordinated Cl^–^···HBD motifs in ion-rich domains—persists an order of magnitude longer than its fast component, which is assigned to labile donor–donor contacts in HBD-rich regions. NADES shows a similarly large ratio but with absolutely longer timescales (τ_2_ ≈ 70 ps) (Table S1, Supporting Information), reflecting its dense, multiply connected network. By contrast, Type V thymol:menthol exhibits a modest ratio (∼2.5) (Table S1, Supporting Information) with both components in the sub-20 ps regime, consistent with its sparse, dispersion-dominated network and low dynamic heterogeneity (α_2_,max ≈ 0.5, Table 5).

Panel (d) correlates viscosity with a dynamic heterogeneity index across the DES series, confirming that systems with greater heterogeneity (larger α_2_,max) (Table S1, Supporting Information) systematically exhibit higher viscosities. The correlation implies that slow-relaxing domains, though occupying a minority of the liquid volume, act as topological bottlenecks that disproportionately govern macroscopic transport. Bubble size in this panel is proportional to hydrogen-bond connectivity, illustrating that the most heterogeneous systems are also the most highly connected—heterogeneity and connectivity are coupled consequences of the cooperative hydrogen-bond network.

Panel (e) presents the temperature dependence of the H-bond heterogeneity index for reline, ethaline, and glyceline over the range 280–380 K. Reline maintains strong heterogeneity across the entire temperature window, decreasing only modestly from ∼1.0 at 280 K to ∼0.8 at 380 K. Ethaline, by contrast, transitions toward homogeneous dynamics above ∼340 K, with the heterogeneity index approaching the values typical of simple molecular liquids. This observation has practical significance: the transport anomalies of DES (non-Stokes–Einstein behavior, composition-dependent probe dynamics, decoupling between rotational and translational diffusion^20^) are most pronounced at temperatures near and below Tg but persist well into the normal fluid regime for highly cooperative systems.

### 3.7 Interfacial Hydrogen-Bond Organization

The behavior of DES at interfaces—electrodes, membranes, catalytic surfaces, and extraction boundaries—is governed by hydrogen-bond restructuring that can differ profoundly from the bulk. Figure 9 presents the interfacial hydrogen-bond organization at a model metal electrode.

**Figure 9:**
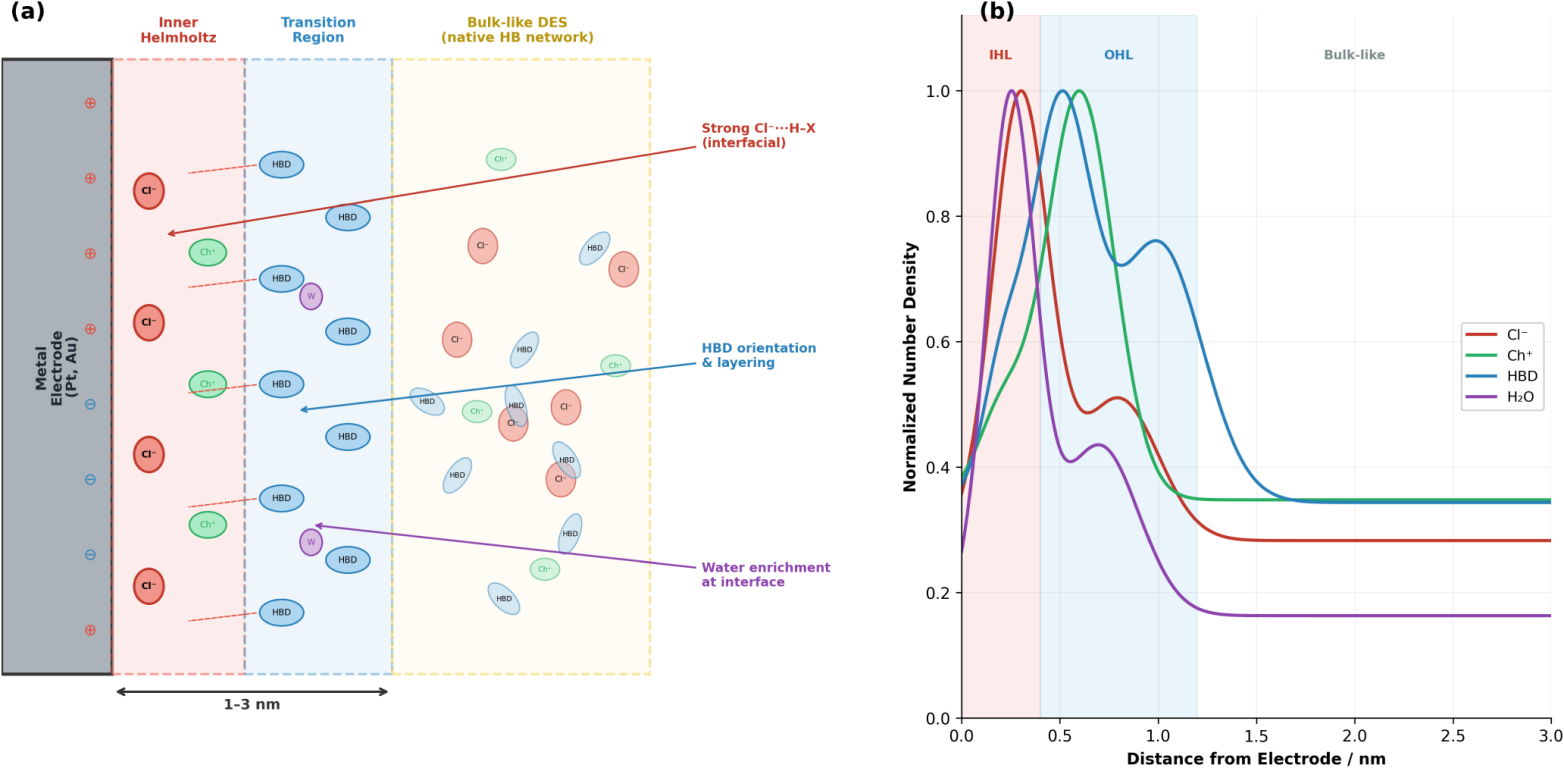
Interfacial hydrogen-bond organization at a metal electrode in a deep eutectic solvent. (a) Schematic of the DES/electrode interface showing the inner Helmholtz layer (IHL), the transition region, and the bulk-like DES zone. Cl^–^ and Ch^+^ alternate near the charged surface, while HBD molecules orient and form layered structures. Water molecules (W) preferentially enrich in the inner layers, competing for Cl^–^ coordination. Red, blue, and purple arrows highlight interfacial ionic H-bonds, HBD orientation and layering, and water enrichment, respectively. (b) Normalized number density profiles for Cl^–^ (red), Ch^+^ (green), HBD (blue), and H_2_O (purple) as a function of distance from the electrode surface. The IHL (pink shading) is characterized by Cl^–^ accumulation, followed by a Ch^+^/HBD-enriched outer Helmholtz layer (OHL, blue shading). Bulk-like composition is recovered beyond ∼2 nm. These profiles illustrate that the interfacial hydrogen-bond network is structurally distinct from the bulk and sensitive to both applied potential and water content.

Panel (a) is a schematic showing three zones: the inner Helmholtz layer (IHL), the transition region, and the bulk-like DES. In the IHL, Cl^–^ accumulates strongly, driven by electrostatic attraction to the electrode surface and by the favorable geometry for forming interfacial Cl^–^···H–X bonds with surface-oriented HBD molecules. Ch^+^ and HBD alternate in the outer Helmholtz layer, creating a layered structure reminiscent of ionic-liquid double layers. Water molecules, when present, preferentially enrich in the inner layers, competing for Cl^–^ coordination sites and further modifying the interfacial bonding environment. Red arrows highlight interfacial ionic hydrogen bonds, blue arrows indicate HBD orientation and layering, and purple arrows mark water enrichment.^38^

Panel (b) provides normalized number density profiles for Cl^–^ (red), Ch^+^ (green), HBD (blue), and H_2_O (purple) as a function of distance from the electrode surface. The Cl^–^ profile peaks sharply in the IHL (pink shading) at approximately 2× the bulk density (Table S1, entry 62)—a remarkable accumulation that creates a locally concentrated ionic environment within ∼0.5 nm of the surface. The Ch^+^/HBD-enriched outer Helmholtz layer (blue shading) follows at 0.5–1.2 nm. Oscillations in all four profiles persist to ∼2 nm (Table S1, entry 64) before bulk-like composition is recovered. The key quantitative result is that the hydrogen-bond motif populations within the IHL are substantially different from those in the bulk: ionic Cl^–^···H–X motifs are enriched, cation-mediated contacts are enhanced, and HBD self-association is suppressed by the geometric constraint of the surface.

These findings have direct consequences for electrochemical performance. The Cl^–^-enriched IHL creates a high local anion concentration that affects electrode kinetics through the specific adsorption of halides, which can either catalyze or poison surface reactions depending on the metal and potential. The HBD depletion (∼40% relative to bulk) (Table S1, entry 63) means that the immediate electrode environment is more ionic and less molecular than the bulk—an important consideration for electrocatalysis in DES, where the assumption of bulk-like composition at the electrode surface can lead to incorrect mechanistic interpretations.^44^ The descriptor framework recognizes this through descriptor 6 (interfacial HB partitioning, Table 3), classified as aspirational because very few studies have attempted systematic interfacial characterization.

### 3.8 Comparative Fingerprints and Design Rules

The integration of all preceding analyses into a comparative and actionable framework is the subject of Figures 10 and 11 and Tables 3 and 4.

**Figure 10:**
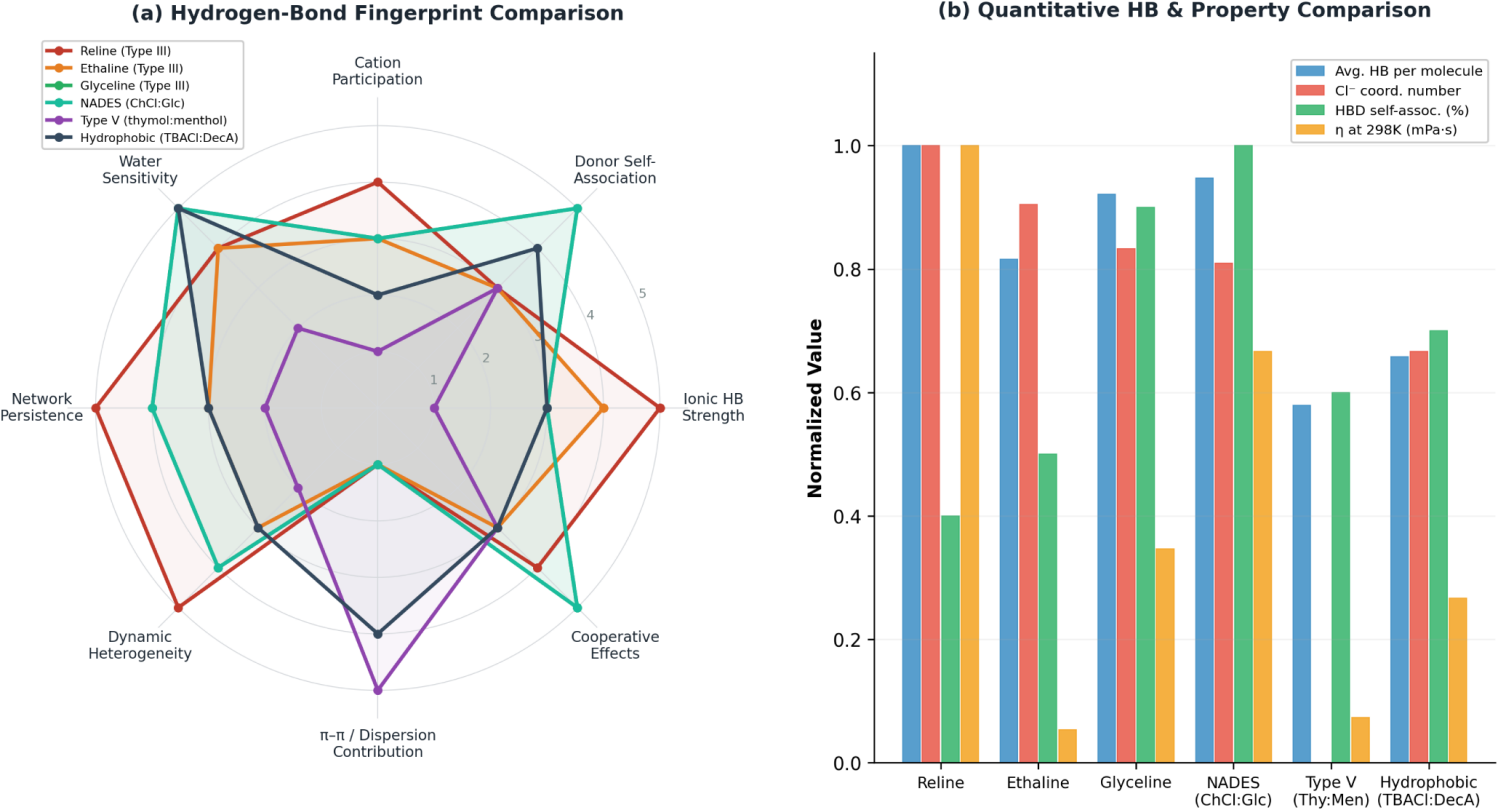
Comparative hydrogen-bond fingerprints across DES types. (a) Radar chart comparing six representative DES systems—reline (Type III), ethaline (Type III), glyceline (Type III), NADES (ChCl:glucose), Type V (thymol:menthol), and hydrophobic (TBACl:decanoic acid)—across eight hydrogen-bond descriptors: cation participation, donor self-association, ionic H-bond strength, cooperative effects, π–π/dispersion contribution, dynamic heterogeneity, network persistence, and water sensitivity. The distinct fingerprint profiles highlight that each DES type possesses a unique hydrogen-bond landscape: reline is dominated by strong ionic motifs and high persistence; glyceline and NADES show pronounced donor self-association and cooperativity; Type V systems are characterized by π–π and dispersion contributions with weak ionic character. (b) Quantitative comparison of normalized hydrogen-bond and property descriptors (average HB per molecule, Cl^–^ coordination number, HBD self-association fraction, and viscosity at 298 K) for the same systems, reinforcing the structure–property relationships discussed throughout this study.

**Figure 11:**
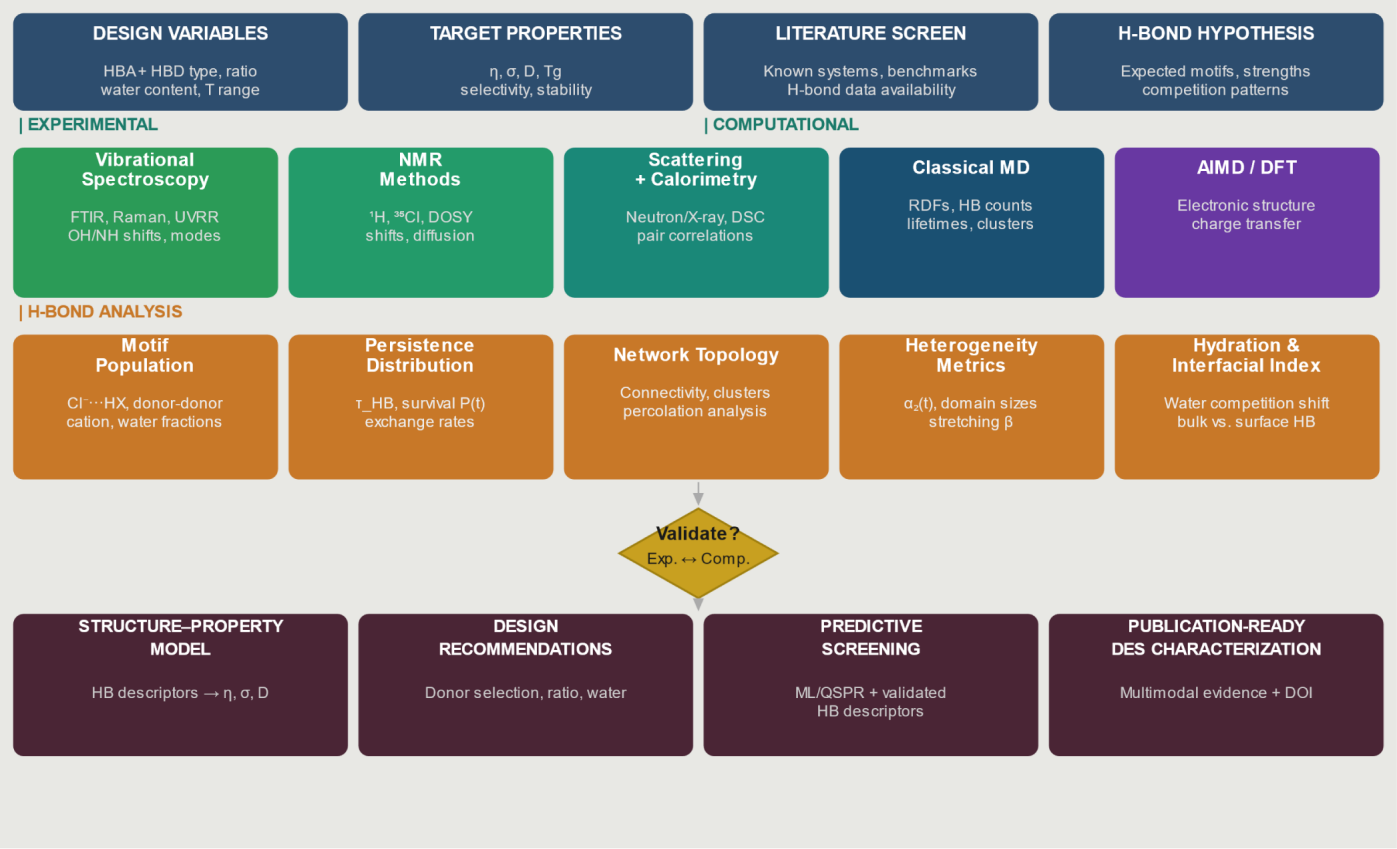
Integrated workflow for DES hydrogen-bond characterization and predictive design. The framework proceeds from initial design variables (HBA/HBD selection, molar ratio, water content, temperature range) and target properties (η, σ, *D*, Tg, selectivity) through a literature screen and H-bond hypothesis, into parallel experimental (vibrational spectroscopy, NMR, scattering/calorimetry) and computational (classical MD, AIMD/DFT) characterization. Results feed into five hydrogen-bond analysis modules: motif population, persistence distribution, network topology, heterogeneity metrics, and hydration/interfacial indices. A central validation step enforces consistency between experimental and computational findings; inconsistent results trigger iterative refinement of force-field parameters and hydrogen-bond definitions. Upon validation, the workflow yields four outputs: a structure–property model linking H-bond descriptors to macroscopic observables, design recommendations for donor/ratio/water optimization, predictive screening via ML/QSPR with validated descriptors, and publication-ready multimodal DES characterization.

Figure 10 introduces the hydrogen-bond fingerprint concept as a tool for DES classification and rational selection. Panel (a) presents a radar chart comparing six representative systems—reline (Type III), ethaline (Type III), glyceline (Type III), NADES (ChCl:glucose), Type V (thymol:menthol), and hydrophobic (TBACl:decanoic acid)—across eight descriptors: cation participation, donor self-association, ionic H-bond strength, cooperative effects, π–π/dispersion contribution, dynamic heterogeneity, network persistence, and water sensitivity. Each system produces a visually distinct fingerprint that encapsulates its hydrogen-bond character in a single, interpretable graphic.

The fingerprints reveal relationships not readily apparent from individual property tables. Reline and NADES share high scores on ionic HB strength and cooperativity but diverge sharply on donor self-association (moderate vs high) and water sensitivity (strong vs very high). Ethaline and glyceline both belong to Type III but differ in network persistence (moderate vs high) and cooperativity (moderate vs high), explaining why glyceline (η ≈ 376 mPa·s) is 10 times more viscous than ethaline (η ≈ 36 mPa·s) despite having the same HBA:HBD ratio.

Panel (b) of Figure 10 provides a quantitative bar chart comparison of four normalized descriptors—average HB per molecule, Cl^–^ coordination number, HBD self-association fraction, and viscosity—for the same six systems. The visual reveals that the HBD self-association fraction tracks more closely with viscosity than does the Cl^–^ coordination number: glyceline and NADES, which exhibit the highest self-association fractions (55% and 65%), also occupy the upper viscosity range, while ethaline (self-association ∼35%) combines moderate Cl^–^ coordination with moderate viscosity.

Figure 11 presents the integrated workflow for DES hydrogen-bond characterization and predictive design that synthesizes the analytical framework of Section 2 with the structure–property insights of Section 3. Stages 1–3—design-variable specification, literature screening, and parallel experimental/computational characterization guided by the method matrix (Figure 3, Table 1)—are detailed in Section 2. Stage 4 feeds the results into five hydrogen-bond analysis modules corresponding to descriptors 1–5 of Table 3: motif population, persistence distribution, network topology, heterogeneity metrics, and hydration/interfacial indices.

A central validation step (depicted as a diamond decision node in Figure 11) enforces consistency between experimental and computational findings. If experiment and simulation agree within predefined tolerances—e.g., ±15% on motif populations, ±25% on lifetimes—the results proceed to output. If inconsistencies are detected, the workflow loops back to refine force-field parameters (charge scaling, LJ parameters) or hydrogen-bond definitions (distance/angle cutoffs), a process that may iterate several times before convergence. This iterative refinement loop is essential for building reliable structure–property models and distinguishes a rigorous characterization campaign from a single-method study.

Upon validation, the workflow yields four outputs: (1) a structure–property model linking hydrogen-bond descriptors to macroscopic observables (Section 3.4, Figure 6); (2) design recommendations for donor selection, molar ratio optimization, and water content control based on the cooperativity–mobility tradeoff (Section 3.3, Figure 5c); (3) predictive screening via ML/QSPR using the validated descriptor set (Table 3) as input features; and (4) publication-ready, multimodal DES characterization meeting the reporting standards of Table 4 (Figures 6 –11).

## 4 Conclusions

This study introduces competitive hydrogen-bond partitioning as an organizing framework for understanding, characterizing, and ultimately designing deep eutectic solvents. Evidence converges from vibrational spectroscopy, multinuclear NMR, neutron and X-ray scattering, classical and ab initio molecular dynamics, DFT cluster calculations, and machine learning potentials.

Five principal conclusions emerge. **First**, the conventional binary model of DES formation is insufficient: the liquid state is better described as a multimotif competition among ionic, donor–donor, cation-mediated, and water-competitive interactions drawing on overlapping donor and acceptor pools, with the equilibrium motif distribution governing macroscopic behavior. **Second**, the cooperativity–mobility tradeoff resolves the long-noted paradox of very low melting points coexisting with very high viscosities: cooperative charge spreading at the chloride solvation shell simultaneously deepens eutectic depression and stiffens the network, defining a design axis from low-cooperativity (Type V) through balanced (ethaline) to high-cooperativity (reline, NADES) systems. **Third**, structure–property correlations are irreducibly multivariate: viscosity tracks with network connectivity, conductivity with the inverse of lifetime, glass transition temperature with persistence, and dynamic heterogeneity with the coexistence of slow ion-rich and fast HBD-rich domains—no single descriptor captures all four, motivating the six-descriptor framework of Table 3. **Fourth**, water is never passive: four hydration regimes are identified in hydrophilic DES (trace, competitive, network-reorganization, and aqueous), the transition from DES to electrolyte is continuous rather than abrupt, and hydrophobic DES are structurally disrupted at just 1–3 wt% moisture. **Fifth**, the interfacial hydrogen-bond environment is structurally distinct from the bulk: Cl^–^ enrichment of ∼2× bulk density and ∼40% HBD depletion in the inner Helmholtz layer create an interfacial motif landscape that governs electrode kinetics and catalytic selectivity, yet remains grossly undercharacterized.

On the basis of these conclusions, four priorities for this field are identified. (i) **Adoption of the Triangulation Criterion**: hydrogen-bond assignments must be supported by at least two independent method categories, one structural and one dynamic, to avoid the overinterpretation endemic to single-technique inferences. (ii) **Standardized reporting**: the 10 minimum standards of Table 4, starting with mandatory water-content determination and force-field provenance, would dramatically improve cross-study comparability and enable data aggregation for machine-learning models. (iii) **Machine-learning force fields** coupled with the validated descriptor set (Table 3) as ML/QSPR input features, to transform DES formulation from an empirical art into a predictive science. (iv) **Systematic interfacial characterization**: combining surface-sensitive spectroscopy (SFG, PM-IRRAS) with interfacial MD and the interfacial partitioning descriptor for any DES application at solid–liquid, liquid–liquid, or liquid–gas boundaries.

The competitive hydrogen-bond partitioning framework and the integrated characterization workflow developed here provide the conceptual and quantitative scaffolding needed to connect molecular-level hydrogen-bond information to the macroscopic properties that ultimately determine whether a DES is fit for purpose.

## Supplementary Material





## Notes

A software tool is developed, allowing a detailed analysis of hydrogen bonding in archetypical DES via suitable properties and developed schemes (ZIP)
Table S1: literature sources and analysis for the considered data (PDF)
