# Supplementary material for: Competitive Hydrogen-Bond Partitioning in Deep Eutectic Solvents: From Cooperative Charge Spreading to Structure–Property Design Rules
Source: ACS Omega. 2026 Jun 22;11(26):38868–91. doi: 10.1021/acsomega.6c02376 (PMC13347335; doi:10.1021/acsomega.6c02376)
Supplement: Supplementary file 1 [file ao6c02376_si_001.zip › des_hb_analyzer.html]

DES Hydrogen-Bond Analyzer


Supporting Tool

# DES Hydrogen-Bond Analyzer

Competitive Partitioning · Cooperativity · Design Rules

Aparicio et al. 2026
