# Supplementary material for: Competitive Hydrogen-Bond Partitioning in Deep Eutectic Solvents: From Cooperative Charge Spreading to Structure–Property Design Rules
Source: ACS Omega. 2026 Jun 22;11(26):38868–91. doi: 10.1021/acsomega.6c02376 (PMC13347335; doi:10.1021/acsomega.6c02376)
Supplement: Supplementary file 2 [file ao6c02376_si_002.pdf]

Competitive Hydrogen-Bond Partitioning in Deep Eutectic Solvents: From Cooperative Charge Spreading to Structure–Property Design Rules

Sergio de-la-Huerta-Sainz,[a] Valentín Diez-Cabanes,[a,b] Alberto Gutiérrez,[a] Sara Santamaría,[a] María A. Escobedo-Monge,[a,b] Pedro A. Marcos,[c] Alfredo Bol,[b,c] José L. Trenzado,[d] Mert Atilhan,[e] Santiago Aparicio[a, b*]

[a] Department of Chemistry, University of Burgos, 09001 Burgos, Spain
[b] International Research Centre in Critical Raw Materials-ICCRAM, University of Burgos, 09001 Burgos, Spain
[c] Department of Physics, University of Burgos, 09001 Burgos, Spain
[d] Department of Physics, University of Las Palmas de Gran Canaria, 35017 Las Palmas de Gran Canaria, Spain
[e] Department of Chemical and Paper Engineering, Western Michigan University, Kalamazoo MI 49008-5462, USA
Corresponding author: sapar@ubu.es

Supporting Information

Table S1. Source Data for All Numerical Ranges Cited in the Main Text

This table provides the primary source documentation for every numerical range or representative value cited in the main text. For each entry, the originating section of the main text is given, followed by the parameter definition, the DES system(s) for which the value was reported, the value or range itself, the method by which it was determined, and the bibliographic reference(s). Where a range reflects our synthesis across multiple independent sources, each source is listed. Entries labelled 'Table 5 (this work)' indicate values assembled by the authors from the cited literature as reported in Table 5 of the manuscript; the individual contributing references for those values are given in the table note to Table 5. Entries where a range is an 'estimate' or 'representative' indicate values that are illustrative syntheses rather than direct measurements; these are so labelled in the corresponding figure captions.

Entry	Section	Parameter	DES system	Value / Range	Method	Ref(s)
1	2.1 / 3.1	HB interaction energy – Cl^-···H–X ionic bonds (overall range)	All Type III	20–55 kJ mol^{-1}	DFT cluster / AIMD	13
2	2.1 / 3.1	HB interaction energy – C–H···Cl^- cation-mediated contacts	All Type III	5–15 kJ mol^{-1}	DFT cluster	13
3	2.1 / 3.2	Effect of charge scaling (0.8×) on computed viscosity	Reline, ethaline, glyceline	Factor of 2–5 improvement	Classical MD comparison	28, 29
4	2.1 / 3.2	Effect of HB geometric cutoff (±0.2 Å, ±10°) on HB counts	Various Type III	20–40% variation	Classical MD sensitivity test	28, 40
5	2.4	Effect of 0.5 wt% residual water on viscosity	Reline	10–20% viscosity shift	Experiment + MD	36, 39
6	2.4	Overestimation of viscosity by unscaled (q = ±1.0) FF	Various	Factors of 3–10	Classical MD vs. experiment	29
7	3.1 / Fig.4	HB interaction energy – Cl^-···H–N ionic bond	Reline (ChCl:urea)	25–55 kJ mol^{-1}	DFT cluster (NBO)	13
8	3.1 / Fig.4	HB interaction energy – Cl^-···H–O ionic bond	Ethaline, glyceline	20–50 kJ mol^{-1}	DFT cluster	13, 31
9	3.1 / Fig.4	HB interaction energy – doubly ionic Ch^+–OH···Cl^-	Reline	30–65 kJ mol^{-1}	DFT cluster (NBO)	13
10	3.1 / Fig.4	HB interaction energy – O–H···O donor self-association	Ethaline, glyceline	15–30 kJ mol^{-1}	DFT cluster / AIMD	30, 31
11	3.1 / Fig.4	HB interaction energy – N–H···O=C urea chains	Reline	18–35 kJ mol^{-1}	DFT cluster	13, 31
12	3.1 / Fig.4	HB interaction energy – C–H···Cl^- (weak)	All Type III	5–15 kJ mol^{-1}	DFT cluster	13
13	3.1 / Fig.4	HB interaction energy – O–H···OH_2 water-competitive	Hydrated DES	12–28 kJ mol^{-1}	AIMD / DFT cluster	30, 31
14	3.1 / Fig.4	Non-HB interaction energy – π–π stacking	Type V (thymol:menthol)	8–25 kJ mol^{-1}	DFT + dispersion correction	21
15	3.1 / Fig.4	Non-HB interaction energy – dispersion	Type V	2–10 kJ mol^{-1}	DFT-D3 / AIMD	21, 30
16	3.1 / Tab.2	Viscosity range across DES families (298 K)	Type V to NADES	~25 to >8000 mPa·s	Rheometry / viscometry	6, 7, 28, 29

17	3.1 / Tab.2	Cl^- coordination number – reline	Reline	3.5–4.5	AIMD / classical MD / neutron diffraction	13, 14, 22
18	3.1 / Tab.2	Dominant HB lifetime – reline and NADES (τ_{HB})	Reline, ChCl:glucose	60–120 ps	Classical MD autocorrelation	17, 22, 33
19	3.1 / Tab.2	Water sensitivity threshold – hydrophobic DES	TBACl:decanoic acid, menthol:decanoic acid	1–3 wt% H_2O	Experiment + MD	37
20	3.1 / Tab.2	Water sensitivity threshold – hydrophilic DES	ChCl-based systems	5–15 wt% H_2O	Neutron diffraction + MD	36
21	3.1 / Tab.2	Tg range – high-connectivity ionic systems	Reline, glyceline, NADES	190–215 K	DSC	Table 5 (this work)
22	3.1 / Tab.2	Tg range – low-connectivity molecular systems	Type V, hydrophobic	145–170 K	DSC	Table 5 (this work)
23	3.3	Charge transfer in isolated $Cl^-\cdots H–N$ pair (Δq)	Reline (gas phase)	≈ 0.03 e	AIMD / NBO analysis	41
24	3.3	Interaction energy of isolated $Cl^-\cdots H–N$ pair (E_{HB})	Reline (gas phase)	≈ −35 kJ mol^{-1}	DFT cluster / NBO	13, 41
25	3.3	Cumulative charge transfer in full solvation shell (Σ Δq)	Reline (liquid)	≈ 0.13 e	AIMD / NBO	13, 41
26	3.3	Total interaction energy in full solvation shell (Σ E_{HB})	Reline (liquid)	≈ −160 kJ mol^{-1}	DFT cluster (many-body)	13
27	3.3	Cooperativity excess over sum of pairwise contributions	Reline	15–40%	DFT many-body cluster	13
28	3.3	Effective charge on Cl^- – isolated ion	Reline	≈ −0.85 e	NBO analysis	41
29	3.3	Effective charge on Cl^- – coordinated (full shell)	Reline	≈ −0.72 e	NBO analysis / AIMD	41
30	3.3 / Fig.5	Cooperative incremental HB energies (1st, 2nd, 3rd donor)	Reline-like systems	−35, −42, −45 kJ mol^{-1}	DFT cluster (representative)	13, 31, 41
31	3.3 / Fig.5	Anticooperative incremental HB energies (1st, 2nd, 3rd donor)	Sterically crowded systems	−28, −18, −12 kJ mol^{-1}	DFT cluster (representative)	13, 31, 41
32	3.3 / Fig.5	τ_{HB} for reline (viscosity paradox discussion)	Reline	60–90 ps	Classical MD	17, 33
33	3.4 / Tab.5	Viscosity data range across Table 5 systems	11 DES systems	8 to >8000 mPa·s	Rheometry / viscometry	Table 5 (this work)
34	3.4 / Tab.5	Conductivity data range across Table 5 systems	11 DES systems	0.01–25 mS cm^{-1}	Impedance spectroscopy	Table 5 (this work)
35	3.4 / Tab.5	Self-diffusion data range across Table 5 systems	11 DES systems	0.1–12 × 10^{-10} m^2 s^{-1}	DOSY NMR / MD	Table 5 (this work)
36	3.4 / Tab.5	HB per molecule range (Table 5)	11 DES systems	1.8–6.5	Classical MD	Table 5 (this work)
37	3.4 / Tab.5	τ_{HB} range (Table 5)	11 DES systems	8–120 ps	Classical MD	Table 5 (this work)
38	3.4 / Tab.5	α_2,max range (Table 5)	11 DES systems	0.3–2.5	Classical MD	Table 5 (this

						work); ref 17
39	3.4	Reline τ_HB (conductivity correlation)	Reline	≈ 75 ps	Classical MD	33
40	3.4	Hydrated DES τ_HB at 30 wt% H_2O	Reline + 30 wt% H_2O	≈ 15 ps	Classical MD / AIMD	36, 43
41	3.4	Tg increases per unit τ_HB (linear trend)	DES series	~1 K per 1 ps	Estimated from Table 5 trend	Table 5 (this work)
42	3.5	Viscosity decrease at competitive hydration (3–15 wt%)	ChCl-based systems	Up to 50%	Experiment + MD	36, 39
43	3.5 / Tab.5	Reline: viscosity at 10 wt% H_2O	Reline	~80 mPa·s (vs ~750 neat)	Experiment + MD	36, 39, Table 5
44	3.5 / Tab.5	Reline: conductivity at 10 wt% H_2O	Reline	~3.5 mS cm^{-1} (vs ~0.2 neat)	Impedance spectroscopy	36, 39, Table 5
45	3.5 / Tab.5	Reline: Cl^- coord. no. shift at 10 wt% H_2O	Reline	3.5–4.5 → 2.8–3.5	MD / AIMD	35, 36, Table 5
46	3.5 / Tab.5	Reline: HB/mol at 10 wt% H_2O	Reline	5.2 → 4.0	Classical MD	Table 5 (this work)
47	3.5 / Tab.5	Reline: α_2,max at 10 wt% H_2O	Reline	2.0 → 1.0	Classical MD	Table 5 (this work); ref 17
48	3.5 / Tab.5	Reline + 30 wt%: full property set	Reline	η≈8 mPa·s; σ≈25 mS cm^{-1}; D≈12×10^{-10} m^2 s^{-1}; α_2,max≈0.3	Experiment + MD	36, 43, Table 5
49	3.5 / Tab.5	HBD self-association collapse at 30 wt% H_2O	Reline	~20% → ~5%	Classical MD	Table 5 (this work)
50	3.5 / Tab.3	Competitive hydration index – reline	Reline	Δ_ionic ≈ −15% at 10 wt%	Classical MD motif counting	Table 3 (this work); ref 36
51	3.5 / Tab.3	Competitive hydration index – hydrophobic DES	TBACl:decanoic acid	Δ_ionic ≈ −60% at 3 wt%	Classical MD motif counting	Table 3 (this work); ref 37
52	3.5 / Tab.3	Competitive hydration index – ethaline	Ethaline (ChCl:EG)	Δ_ionic ≈ −8% at 10 wt%	Classical MD motif counting	Estimated; refs 24, 32
53	3.6	α_2,max values for ChCl-based systems	ChCl-based DES series	~0.5 (dilute) to ~2.5 (neat NADES)	Classical MD	17
54	3.6 / Fig.8	Reline HB survival: stretching exponent β and τ_HB	Reline	β ≈ 0.55; τ_HB ≈ 75 ps	Classical MD autocorrelation	33
55	3.6 / Fig.8	Ethaline HB survival: β and τ_HB	Ethaline	β ≈ 0.65; τ_HB ≈ 32 ps	Classical MD autocorrelation	33
56	3.6 / Fig.8	Domain exchange timescale	Reline, glyceline	50–200 ps	Classical MD (spatial density)	17, 33, 34
57	3.6 / Fig.8	Individual HB breaking timescale	All DES	~5–20 ps	AIMD / classical MD	22, 28, 33

58	3.6 / Fig.8	Reline τ_2/τ_1 relaxation ratio	Reline	~10	Classical MD	33
59	3.6 / Fig.8	NADES slow component τ_2	ChCl:glucose	≈ 70 ps	Classical MD	Table 5 (this work)
60	3.6 / Fig.8	Type V τ_2/τ_1 ratio	Thymol:menthol	~2.5	Classical MD	Estimated from Table 5
61	3.6 / Fig.8	Nanoscale domain spatial scale	All DES	1–5 nm	Neutron diff. + MD	14, 17, 34
62	3.7 / Fig.9	Cl^- enrichment at inner Helmholtz layer	Reline at Pt electrode	~2× bulk density	Neutron reflectometry + MD	38
63	3.7 / Fig.9	HBD depletion at inner Helmholtz layer	Reline at Pt electrode	~40% relative to bulk	Neutron reflectometry + MD	38
64	3.7 / Fig.9	Bulk-like composition recovery distance from electrode	Reline	~2 nm	MD number density profiles	38

Note on scope: This table covers all quantitative ranges appearing in the body text, figures, and their captions. Values that appear exclusively within Table 2, Table 3, Table 5, or Table 6 of the main manuscript are documented at the table level through the reference columns of those tables and are not repeated here unless they are also cited individually in the text. Ranges appearing in figure captions only are included here where they contain independent numerical claims not also stated in the running text.
